# Genes That Act Downstream of Sensory Neurons to Influence Longevity, Dauer Formation, and Pathogen Responses in *Caenorhabditis elegans*

**DOI:** 10.1371/journal.pgen.1003133

**Published:** 2012-12-20

**Authors:** Marta M. Gaglia, Dae-Eun Jeong, Eun-A Ryu, Dongyeop Lee, Cynthia Kenyon, Seung-Jae Lee

**Affiliations:** 1 Neuroscience Graduate Program and Department of Biochemistry and Biophysics, University of California San Francisco, San Francisco, California, United States of America; 2 Division of Molecular and Life Sciences/I-BIO/World Class University Program IT Convergence Engineering, Pohang University of Science and Technology, Pohang, South Korea; Harvard University, United States of America

## Abstract

The sensory systems of multicellular organisms are designed to provide information about the environment and thus elicit appropriate changes in physiology and behavior. In the nematode *Caenorhabditis elegans*, sensory neurons affect the decision to arrest during development in a diapause state, the dauer larva, and modulate the lifespan of the animals in adulthood. However, the mechanisms underlying these effects are incompletely understood. Using whole-genome microarray analysis, we identified transcripts whose levels are altered by mutations in the intraflagellar transport protein *daf-10*, which result in impaired development and function of many sensory neurons in *C. elegans*. In agreement with existing genetic data, the expression of genes regulated by the transcription factor DAF-16/FOXO was affected by *daf-10* mutations. In addition, we found altered expression of transcriptional targets of the DAF-12/nuclear hormone receptor in the *daf-10* mutants and showed that this pathway influences specifically the dauer formation phenotype of these animals. Unexpectedly, pathogen-responsive genes were repressed in *daf-10* mutant animals, and these sensory mutants exhibited altered susceptibility to and behavioral avoidance of bacterial pathogens. Moreover, we found that a solute transporter gene *mct-1/2*, which was induced by *daf-10* mutations, was necessary and sufficient for longevity. Thus, sensory input seems to influence an extensive transcriptional network that modulates basic biological processes in *C. elegans*. This situation is reminiscent of the complex regulation of physiology by the mammalian hypothalamus, which also receives innervations from sensory systems, most notably the visual and olfactory systems.

## Author Summary

The senses provide animals with information about their environment, which affects not only their behavior but also their internal state and physiological outputs. How this information is processed is still unclear. In this study, we used mutant *C. elegans* roundworms that had defective sensory neurons to investigate how changes in sensation alter the expression of genes and regulate physiology, specifically the worms' choice to hibernate during growth and their longevity as fully-grown adults. We showed that defects in sensory neurons change the pattern of gene expression and regulate these outputs through known hormonal pathways, including insulin/IGF-1 and steroid pathways. We also identified a new regulator of longevity, MCT-1, that is predicted to transport small metabolites and hormones in the body. Unexpectedly, we found that sensory impairment altered yet another physiological output, the response to infectious agents. It prevented the worms from avoiding infectious bacteria and reduced the expression of potentially protective factors, but also increased the worms' resistance to infection, suggesting a complex network of responses to environmental stimuli. Understanding how sensory information is relayed in this relatively simple organism may inform our understanding of sensory processing in higher organisms like mammals.

## Introduction

Organisms are constantly interacting with their environment. Behavioral responses as well as physiological processes such as energy homeostasis, development and immune homeostasis need to be modulated depending on the environmental situation. For example, an animal's feeding and development are crucial to survival in general, but may need to be reduced or delayed under certain environmental conditions to allow efficient allocation of resources for survival. To achieve such modulation, animals have developed complex sensory systems that acquire and integrate various sorts of information about their environment and their internal state. However, the mechanisms by which sensory neurons influence complex physiological processes are still incompletely understood.

Because of its relatively simple nervous system and genetic tractability, the nematode *Caenorhabditis elegans* has been studied extensively as an experimental organism to dissect the molecular mechanisms regulating sensory control of behavior. A small number of ciliated sensory neurons located mainly near the head and tail of the animal detect environmental signals, including soluble and volatile compounds, gases, osmolarity, and mechanosensory and noxious stimuli (reviewed in [1]). As in other organisms, it is now clear that the sensory system of *C. elegans* regulates physiological functions of the animals as well as its behavior. When certain sensory neurons are compromised, for example, worms are more likely to arrest in an alternative developmental state of diapause called dauer in response to higher temperatures [2]–[4]. In turn, dauer arrest results in modulation of behavioral output and reduced response to stimuli [5]. In addition, various signaling pathways in the nervous system contribute to lifespan regulation [3], [6]–[8] and the response to pathogenic insults [9]–[13]. One possible model is that sensory input is translated into whole-organism physiological alterations by the regulation of transcriptional programs. However, to date only a few genes have been reported to regulate physiological changes downstream of sensory perception, most notably the Forkhead transcription factor *daf-16*/FOXO [3], [14]–[16], an important regulator of lifespan, dauer formation and immune response downstream of the *daf-2*/insulin/IGF1-like receptor (InsR). Thus, much remains to be learned about how the disruption of sensory neurons results in changes in the physiology of the whole organism.

In this study, we examine the transcriptional profile of *daf-10* sensory mutants to identify genes and signaling pathways that may be targeted by the sensory system to regulate the physiology of the animal. The *daf-10* gene encodes the *C. elegans* homolog of the intraflagellar transport protein IFT222 and its mutation results in altered development of a number of sensory neurons [17], [18], leading to defects in sensory perception. *daf-10* mutants also exhibit an increase in longevity and an increase in spontaneous dauer formation at high temperature (27°C) [2], [3]. In *daf-10* mutant animals we find evidence of activation of DAF-16/FOXO, as expected, as well as activation of the nuclear hormone receptor (NHR) DAF-12, which we demonstrate is required for the increased dauer formation, but not for the extended longevity of sensory mutant animals. In addition, we show that the response to pathogenic bacteria is altered in the sensory *daf-10* mutant animals and that both behavioral and physiological responses to pathogens are affected. Furthermore, we examine the functional significance of genes that are up-regulated by *daf-10* mutations and find that *mct-1*/*2*, a putative monocarboxylate transporter, is required for the extended lifespan of *daf-10* mutants. Taken together, our data suggest that the sensory system modulates several transcriptional programs to exert its effects on lifespan, dauer formation and innate immunity in response to the environmental changes.

## Results

### Microarray analysis reveals differentially expressed genes in the long-lived *daf-10(m79)* mutant

To identify the genes and pathways that are downstream of the sensory system, we used whole-genome oligonucleotide-based microarrays to compare gene expression in young adult animals of a long-lived sensory mutant strain, *daf-10(m79)*, with that of wild-type young adult worms. Many of the mutations that compromise the development and function of the sensory neurons of *C. elegans* also lead to altered longevity and dauer formation at high temperature [3], [8], [16]. We chose this particular mutant strain because *daf-10* has been shown to be expressed in all ciliated neurons [19] and multiple alleles of *daf-10* cause a significant longevity phenotype [3], showing that the effect of *daf-10* sensory mutations is robust.

**Figure 1:**
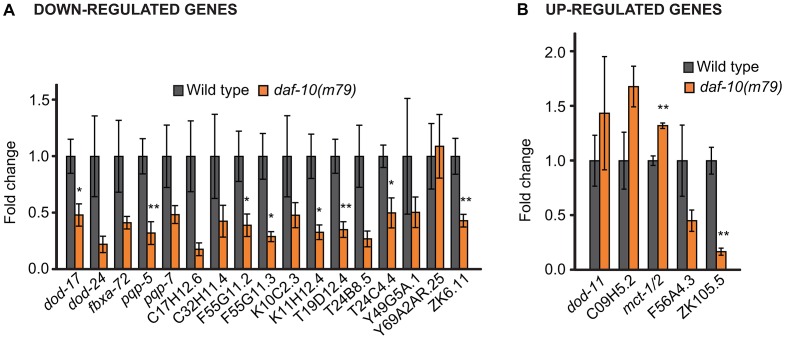
qRT–PCR analysis confirms differential regulation by *daf-10(m79)* mutations of genes identified through microarray analysis. qRT-PCR was used to examine changes in the expression of genes that were down-regulated (A) or up-regulated (B) in the microarray analysis. Error bars represent s.e.m. (* *p*<0.05, ** *p*<0.01, Student's *t-*test).

Our microarray analysis identified 14 genes that were reliably up-regulated in the *daf-10(m79)* animals and 56 genes that were down-regulated (Table 1 and Table 2). We further re-tested 5 of the up-regulated and 17 of the down-regulated genes using quantitative RT-PCR (qRT-PCR) analysis and confirmed that 18 out of the 22 genes that we tested showed similar changes using qRT-PCR and microarray analysis (Figure 1A and 1B). In addition, our qRT-PCR analysis showed that 9 out of the 22 genes exhibited a similar trend in expression in *osm-5(p813)*, another mutant with defective ciliated neurons and extended longevity [3] (Figure S1A and S1B). The *osm-5* gene encodes another component of the intraflagellar transport complex [20], [21], which becomes truncated by a premature stop codon in *p813*. Thus, our microarray analysis has identified a small but reliable set of genes that are differentially expressed in response to defects in sensory perception.

**Table 1:** Genes up-regulated in *daf-10(m79)* mutants versus wild-type animals.

Gene	Brief description	*p* value	Fold change	qRT-PCR data	DAF-16 target?	DAF-12 target?	Pathogen-responsive?	Tissue expression?
F56A4.3	Glutathione-S-tranferase	8.20E-05	18.42	Not repeated				
Y19D10A.7/*irld-53*	EGF receptor, L-domain	5.73E-04	6.44			D*, E		Neuronal (enriched, H)
W02D7.8	Unknown	0.003	4.47					Neuronal (expressed, H)
F14D7.7	Unknown	0.003	4.41					
K12G11.3/*dod-11* (*sodh-1*)	Alcohol dehydrogenase	0.006	4.09	Up in *osm-5(p813)*	A, C		F	Intestinal (expressed, I)
ZC84.3/*cls-3*	CLASP-family microtubule binding protein	0.006	3.64					Neuronal (enriched, H)
Y19D10A.12/*mct-1/2*	Monocarboxylate transporter	0.007	3.55	Up in *daf-10(m79)*, down in *osm-5(p813)*		E		
ZK105.5	Unknown, DUF19	0.011	3.26	Not repeated	C			
F53A3.1	Unknown	0.016	3.21					
F45D11.14	Unknown, DUF684	0.015	3.08					
F21C10.11	Unknown	0.016	3.03		C	D*		
C09H5.2/*catp-3*	Cation-transporting ATPase. Regulated by p38 MAP kinase pathway [33]	0.016	2.99	Up in *osm-5(p813)*				
F53B2.2/*tsp-4*	Tetraspanin family integral membrane protein	0.018	2.98					
R08E5.4	Unknown	0.020	2.96		B			

A = up-regulated in a *daf-16-*dependent fashion in *daf-2* mutants, Murphy *et al.*, 2003 [31].

B = up-regulated in a *daf-16-*dependent fashion in *daf-2* mutants, McElwee *et al.*, 2004 [35].

C = up-regulated in a *daf-16-*dependent fashion in *daf-2* mutants, Lee *et al.*, 2009 [34].

D* = down-regulated by DAF-12, Fisher and Lithgow, 2006 [30].

E = up-regulated by DAF-12, Shostak *et al.*, 2004 [36].

F = repressed during PA14 exposure, Shapira *et al.*, 2006 [32].

H = Von Stetina *et al.*, 2007 [23].

(“Expressed”: genes whose expression is detected in the neurons, “Enriched”: genes that are solely or more highly expressed in neurons).

I = Pauli *et al.*, 2006 [22].

(“Expressed”: genes whose expression is detected in the intestine, “Enriched”: genes that are solely or more highly expressed in intestine).

**Table 2:** Genes down-regulated in *daf-10(m79)* mutants versus wild-type animals.

Gene	Brief description	*p* value	Fold change	qRT-PCR data	DAF-16 target?	DAF-12 target?	Pathogen-responsive?	Tissue expression?
T21E8.2/*pgp-7*	P-glycoprotein, ABC transporter	7.97E-05	0.08	Down in *daf-10(m79)*			G	Tail, intestine [67], [68]
C05A9.1/*pgp-5*	P-glycoprotein, ABC transporter	2.44E-04	0.10	Down in *daf-10(m79)*	B		G	Intestine [68]
K01A2.3	Unknown	0.004	0.14					Intestine, muscle [69], [70]
C07G3.2/*irg-1*	Riboflavin synthesis protein, DUF1768	0.003	0.15	Not induced by PA14 in *daf-10(m79)*			F, G	Intestinal (enriched, I)Intestinal, induced by PA14 [71]
B0024.1/*col-149*	Collagen	0.001	0.17					
Y49G5A.1	Serine protease inhibitor	0.002	0.18	Down in *daf-10(m79)*, *osm-5(p813)*Regulated by *daf-12*	B*	D	G*	
W06G6.12	Unknown	0.004	0.20					
T21E8.1/*pgp-6*	P-glycoprotein, ABC transporter	0.002	0.21				G	Neuronal (expressed, H)Neurons, intestine [68]
C32H11.12/*dod-24*	Unknown, DUF141, Age	0.002	0.21	Down in *daf-10(m79)*, *osm-5(p813)*Regulated by *daf-12*	A, B	D	F, G	
T24C4.4	Unknown, DUF1164	0.003	0.23	Down in *daf-10(m79)*Induction by PA14 is reduced in *daf-10(m79)*	B, C		F, G	Intestinal (expressed, I)
C32H11.4	Unknown, DUF141	0.003	0.25	Down in *daf-10(m79)*, *osm-5(p813)*Induction by PA14 is reduced in *daf-10(m79)*Regulated by *daf-12*	A, B, C	D	F, G	
C10H11.6/*ugt-26*	UDP-glucuronoyl transferase	0.005	0.25		B		G	
C03H5.1/*clec-10*	C-type lectin	0.004	0.26		B		F*, G*	Intestinal (expressed, I)
F55G11.2	Unknown, DUF141	0.004	0.26	Down in *daf-10(m79)*, *osm-5(p813)*Regulated by *daf-12*	B, C	D	F, G	
F38A5.9/*nspb-5*	Unknown, worm specific, DUF1459	0.018	0.27					
K10D11.1/*dod-17*	Unknown, DUF141, Age	0.007	0.27	Down in *daf-10(m79)*, *osm-5(p813)*Induction by PA14 is reduced in *daf-10(m79)*Regulated by *daf-12*	A, B, C	D	F, G	Intestinal (expressed, I)
T20D4.3	Peptide N glycanase, worm specific, DUF750	0.009	0.28				G*	
C17H12.6	Unknown, DUF141	0.007	0.29	Down in *daf-10(m79)*, *osm-5(p813)*Regulated by *daf-12*	B	D	F, G	Intestinal (enriched, I)
Y22D7AR.10	Histone acetyltransferase	0.006	0.29					
T20D4.5	Peptide N-glycanase, DUF750	0.008	0.30					
F42A9.6	Unknown, possible function in germline	0.006	0.30					Commonly expressed in germline, muscle and intestine (I)Neuronal (expressed, H)
Y53F4B.11	Unknown	0.011	0.31					
T02E1.7	SURF4 family, putative cargo protein	0.015	0.31					
Y45F10C.2	Unknown, DUF1505	0.007	0.31					Uterus [72]
F35E12.5	Unknown, DUF141	0.008	0.31		A, B, C	D	G	
Y69A2AR.25	Unknown	0.011	0.32	Up in *osm-5(p813)*		D		
C29F9.14/C29F9.9	Unknown, 7TM receptor	0.009	0.32		C*			
ZK6.11	Transmembrane glycoprotein, DUF274, regulates fat accumulation, aging	0.011	0.32	Down in *daf-10(m79)*	A, B, C		G	Intestinal (enriched, I)
F19B2.5	Helicase-like transcription factor	0.013	0.33				F, G	Intestinal (expressed, I)
C29F9.3	Unknown	0.010	0.33					Intestinal (enriched, I)
F55G11.3	Pseudogene	0.014	0.34	Down in *daf-10(m79)*, *osm-5(p813)*Regulated by *daf-12*	B	D	G	
F31F4.15/*fbxa-72*	F-box protein	0.017	0.34	Down in *daf-10(m79)*, *osm-5(p813)*Regulated by *daf-12*	B	D	G*	Intestinal (enriched, I)
C08F11.12	Unknown, DUF1505	0.015	0.35					
K11H12.4	Unknown, DUF274, predicted GPI-anchor	0.017	0.35	Not repeated	B, C		G	
C17H12.8	Unknown, DUF141	0.016	0.37		A		G	Intestinal (enriched, I)
C14C6.5	ShK toxin domain	0.015	0.38				F	Intestinal (expressed, I)
D1025.6/*nspc-16*	Unknown, worm specific	0.015	0.38					
C33C12.4	Unknown, worm specific, Emb	0.020	0.38					Intestinal (expressed, I)
Y58A7A.3	RNAse III family, Zn-finger protein	0.018	0.39		B		G	
T19D12.4	Unknown, Esp	0.017	0.40	Down in *daf-10(m79)*	A. B		F, G	Intestinal (enriched, I)
Y69A2AR.13	Unknown	0.019	0.40					
C29F3.7	Unknown, DUF141, Gro	0.020	0.44		B		F, G	Intestinal (enriched, I)
T24B8.5	ShK toxin domain	0.019	0.56	Down in *daf-10(m79)*, *osm-5(p813)*	A, B		F	
R06C1.4	RNA binding protein	0.019	0.56		B			Intestinal (expressed, I)
C55B7.4/*acdh-1*	Acyl CoA dehydrogenase, Esp, Age	0.018	0.56				F*, G*	Intestinal (expressed, I)
C36C5.5	Worm-specific Cys-rich secreted protein, DUF19	0.019	0.57					
F36D1.4	Unknown	0.019	0.57			D*		
C06G3.12	Transposon	0.019	0.57					
Y69E1A.8	Unknown	0.017	0.57					
F25H5.8	Member of UPF0057, stress responsive protein	0.018	0.57					
F38E1.3	Protein kinase	0.020	0.57					
ZK1251.1/*htas-1*	Histone H2A variant	0.017	0.57			D*		Germline [73]
T22A3.2/*hsp-12.1*	Member of Hsp20 protein family	0.019	0.57					Muscle [69]
K08E7.4	Unknown	0.019	0.57					
K10C2.3	Aspartyl protease	0.020	0.57	Down in *daf-10(m79)*		D	F, G	
C54F6.13/*nhx-3*	Sodium/proton exchanger	0.019	0.69			D		Hypodermis, reproductive system [74]

A = down-regulated in a *daf-16-*dependent fashion in *daf-2* mutants, Murphy *et al.*, 2003 [31].

B = down-regulated in a *daf-16-*dependent fashion in *daf-2* mutants, McElwee *et al.*, 2004 [35].

B* = up-regulated in a *daf-16-*dependent fashion in *daf-2* mutants, McElwee *et al.*, 2004 [35].

C = down-regulated in a *daf-16-*dependent fashion in *daf-2* mutants, Lee *et al.*, 2009 [34].

C* = up-regulated in a *daf-16-*dependent fashion in *daf-2* mutants Lee *et al.*, 2009 [34].

D = down-regulated by DAF-12, Fisher and Lithgow, 2006 [30].

D* = up-regulated by DAF-12, Fisher and Lithgow, 2006 [30].

F = up-regulated during PA14 exposure, Shapira *et al.*, 2006 [32].

F* = down-regulated during PA14 exposure, Shapira *et al.*, 2006 [32].

G = up-regulated during PA14 exposure, Troemel *et al.*, 2006 [33].

G* = down-regulated during PA14 exposure, Troemel *et al.*, 2006 [33].

H = Von Stetina *et al.*, 2007 [23].

(“Expressed”: genes whose expression is detected in the neurons, “Enriched”: genes that are solely or more highly expressed in neurons).

I = Pauli *et al.*, 2006 [22].

(“Expressed”: genes whose expression is detected in the intestine, “Enriched”: genes that are solely or more highly expressed in intestine).

### Comparative analysis of the genes that are differentially expressed in *daf-10(m79)* worms

We used several previously published microarray datasets to determine the tissue-specific expression and the potential function of the genes we had identified as differentially expressed in the *daf-10(m79)* mutant animals. First we compared our gene list to those of genes enriched or solely expressed in intestine [22], neurons [23] or muscle [22]. Of the 70 genes that we identified, 22 are likely expressed in the intestine and 5 in neurons (Table 1 and Table 2). Six genes are expressed in other tissues. We also compared our genes to a *C. elegans* global expression map created using 553 microarray data sets (http://nemates.org/gl/cgi-bin/gene_list.cgi?set=20002) [24]. This map represents the correlation in gene expression mapped against gene density in three dimensions and can be used as a tool to assign gene function based on co-regulation with known sets of genes. The genes differentially regulated in *daf-10(m79)* animals mapped predominantly to mountains 19 and 21. Mountain 19 includes genes that are also changed in response to mutations in the *daf-2*/InsR pathway in a *daf-16*/FOXO-dependent manner or in response to mutations in *daf-12*/NHR. Four out of five of the genes that overlap with mountain 21 may be involved in detoxification: the UDP-glucuronosyltransferase (UGT) *ugt-26* and the P-glycoproteins *pgp-5*, *-6*, *-7*. UGTs metabolize foreign substances and endogenous toxins by glycosylating these molecules and facilitating their elimination. P-glycoproteins are members of the ABC transporter family and function to extrude large hydrophobic molecules from cells [25], [26]. *pgp-5* may also have a role in immune responses in *C. elegans* [27]. Interestingly, classification of the differentially expressed genes by GO-term annotation using the DAVID program [28], [29] also highlighted genes with ATPase or transport activity (which include *pgp* genes), as well as genes involved in aging or determination of lifespan (which include mountain 19 genes). When we used the DAVID program to classify genes based on protein domains as defined by the Interpro database [29], proteins with a CUB-like domain (formerly known as DUF141) emerged as overrepresented. CUB-like domains are *C. elegans*-specific domains that resemble CUB domains, which are found on extracellular and membrane proteins such as complement proteins. CUB-like domains may be found predominantly within secreted proteins [30]. In addition, proteins with a CUB-like domain may have important, though unknown, roles in longevity. Two such proteins, *dod-24* and *dod-17*, are down-regulated in long-lived *daf-2*/InsR mutant animals and knockdown of these genes by RNA interference increases lifespan [31]. CUB-like domain proteins were also identified among the transcriptional targets of *daf-12*/NHR [30] and among genes that are activated by exposure to the pathogen *Pseudomonas aeruginosa* [32], [33] (see below).

Because the extended lifespan of *daf-10(m79)* animals is dependent on the *daf-16*/FOXO transcription factor and because of the mapping to mountain 19, we compared our list with genes whose expression changes in *daf-2* mutants in a *daf-16*/FOXO-dependent fashion. We found a significant overlap between our gene set and three independent DAF-16/FOXO target lists (Table 3, *p*<0.0001 in all three cases, hypergeometric probability) [31], [34], [35]. Specifically, of 70 differentially regulated genes in our array list, 9 were also identified by Murphy et al. [31], 21 by McElwee et al. [35] and 10 by Lee et al. [34]. This is consistent with genetic data that has implicated *daf-*16/FOXO and presumably insulin signaling in the regulation of lifespan downstream of the sensory system [3], [15]. It is notable that the overlaps among these three studies are of comparable size (for example, 36% of the 506 genes identified by Murphy et al. and 23% of the 457 genes identified by Lee at al. were also detected in the study by McElwee et al., compared to 30% of the 70 genes identified in our study). Only 31 genes were identified in all three studies, and of these 4 genes were also identified as differentially regulated in *daf-10(m79)* animals.

**Table 3:** Overlap between the genes differentially regulated in *daf-10(m79)* animals and previous gene expression analysis results.

Paper	Comparison	Total # differentially expressed genes		Up in *daf-10(m79)*?[14 genes]	Down in *daf-10(m79)*?[70 genes]
Murphy et al., 2003 [31]	*daf-2* vs. *daf-16; daf-2*	Up in *daf-2*:Down in *daf-2*:	256250	10	08
McElwee at al., 2004 [35]	*daf-2* vs. *daf-16; daf-2*	Up in *daf-2*:Down in *daf-2*:	1110780	20	119
Lee et al., 2009 [34]	*daf-2* vs. *daf-16; daf-2*	Up in *daf-2*:Down in *daf-2*:	157300	31	27
Fisher and Lithgow, 2006 [30]	*daf-12(gof)* vs. *daf-12(lof)*	Up in *daf-12(gof)*:Down in *daf-12(gof)*:	83142	02	213
Shapira et al., 2006 [32]	PA14-exposed vs. control	Up with PA14:Down with PA14:	19634	01	152
Troemel et al., 2006 [33]	PA14-exposed vs. control	Up with PA14:Down with PA14:	311122	00	195
Troemel et al., 2006 [33]	PA14-exposed (8 hrs) vs. control	Up with PA14:Down with PA14:	271236	00	213

*lof*: loss of function.

*gof*: gain of function.

In summary, unbiased analysis using the *C. elegans* expression map and classification by DAVID as well as directed comparison with *daf-2*/InsR- and *daf-16*/FOXO-regulated genes reveal that the genes differentially regulated in *daf-10(m79)* sensory mutants are likely involved in lifespan regulation and insulin-like signaling in the worm. Additionally, these analyses uncovered the overrepresentation of a second class of genes, those involved in detoxification, in the dataset.

### The sensory system works through *daf-12*/NHR to control dauer formation, but not lifespan

**Figure 2:**
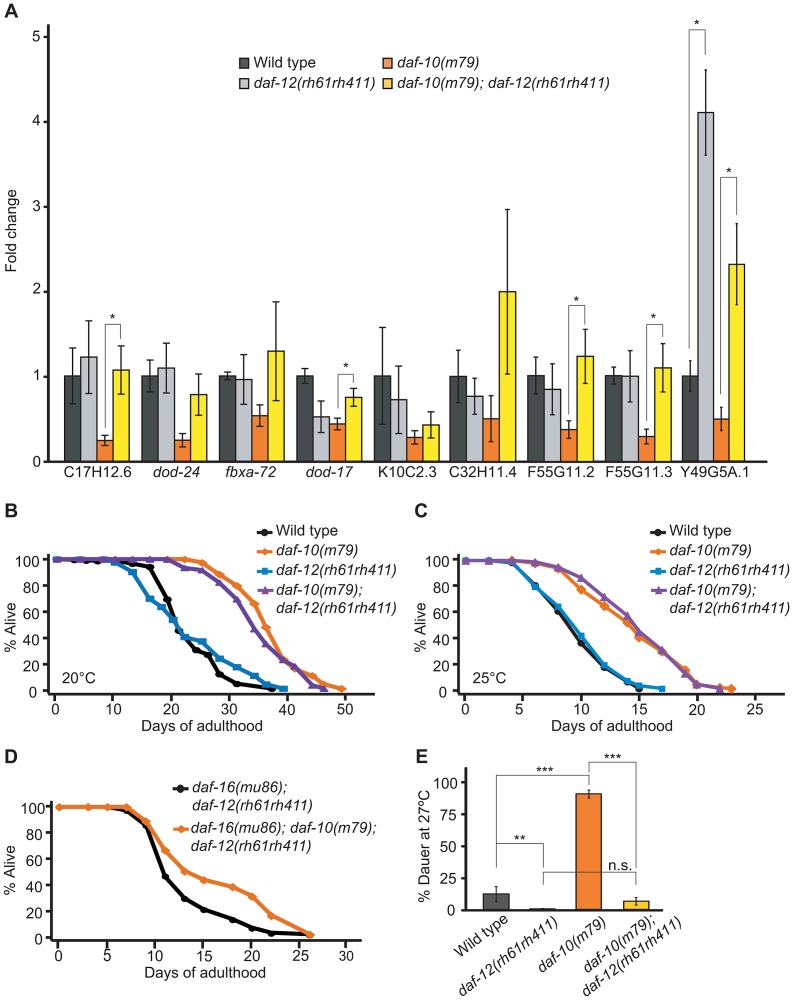
*daf-10* mutations influence the expression of DAF-12/NHR-regulated genes. A. qRT-PCR was used to determine whether the putative DAF-12 targets were regulated in a *daf-12*/NHR-dependent fashion in the *daf-10(m79)* background. B–C. *daf-12*/NHR was not required for *daf-10(m79)* mutant animals to live long at 20°C (B) or at 25°C (C). D. Mutations in *daf-10* can still extend the lifespan of *daf-16(mu86); daf-12(rh61rh411)* mutant animals. A summary of the data presented in these panels and additional repeats is included in Table S1A. E. *daf-12*/NHR was required for *daf-10(m79)* mutants to arrest at the dauer stage when grown at 27°C. Note that the difference between *daf-10(m79); daf-12(rh61rh411)* and *daf-12(rh61rh411)* animals was not statistically significant (*p* = 0.07, n.s.). Error bars represent s.e.m. (* *p*<0.05, ** *p*<0.01, *** *p*<0.001, Student's *t-*test).

Because both putative *daf-12*/NHR targets and genes differentially expressed in *daf-10(m79)* sensory mutant worms mapped to mountain 19 and included a number of CUB-like genes [29], we hypothesized that DAF-12/NHR targets may also be regulated by the sensory system. Therefore, we compared our dataset with a list of putative DAF-12/NHR targets identified in a comparative study of *daf-12*/NHR gain-of-function and loss-of-function mutant animals [30] and found a very significant overlap (Table 3, *p*<0.0001, hypergeometric probability; genes that were reported as DAF-12/NHR-binding target genes in another study [36] are also indicated in Table 1 and Table 2). We then performed qRT-PCR to compare the expression of these putative DAF-12/NHR target genes in the presence and absence of *daf-12*/NHR, both in *daf-10* sensory mutants and in otherwise wild-type animals (Figure 2A). We found that of the nine genes tested, eight were no longer down-regulated by *daf-10* mutations in the *daf-12(rh61rh411)* null mutant background (Figure 2A), confirming that these genes are indeed regulated by DAF-12/NHR in the context of *daf-10* sensory mutations. Interestingly, loss of DAF-12/NHR alone did not alter the expression of most of the genes tested, with the exception of Y49AG5A.1 and *dod-17* (in the latter case, the effect was opposite to what was expected). Together with the microarray results, these data indicate that the activity of DAF-12/NHR is altered as a result of sensory system mutations. Because the basal level of the target genes was largely unchanged in *daf-12(rh61rh411)* animals, DAF-12/NHR is likely to be specifically activated in sensory mutant worms.

Since *daf-12*/NHR is important for both lifespan regulation and development into the arrested dauer state [37], [38] and both of these processes become misregulated in sensory mutants, we tested whether *daf-12*/NHR was required for increased lifespan or dauer formation in *daf-10(m79)* animals. *daf-12*/NHR appeared to be dispensable for the extended longevity of *daf-10(m79)* animals at 20°C (Figure 2B). We previously showed that *daf-12*/NHR was required for the influence of thermosensory neurons on lifespan at 25°C [8]. We therefore tested whether *daf-12*/NHR was required for the longevity caused by *daf-10(m79)* sensory mutation at 25°C, but found it was not (Figure 2C). In addition, since *daf-16(mu86); daf-10(m79)* animals have a small residual increase in lifespan compared to *daf-16(mu86)* animals [3], we also tested specifically whether *daf-12*/NHR was required for the *daf-16*/FOXO-independent portion of the lifespan increase. However, *daf-10* mutations still extended lifespan slightly in the *daf-16(mu86); daf-12(rh61rh411)* background (Figure 2D). In contrast, *daf-12*/NHR was required for the increased high-temperature dauer formation due to *daf-10* mutations (Figure 2E, percentage dauers, mean ± s.e.m.: wild type = 11.8%±5.7%, *daf-12(rh61rh411)* = 0.4%±0.3%, *daf-10(m79)* = 88.8%±3.2%, *daf-10(m79); daf-12(rh61rh411)* = 6.3%±3.0%). This is consistent with what was observed with *tax-4* mutations, which eliminate the function of a cyclic nucleotide-gated channel required for sensory transduction [39] and also cause increased dauer formation at 27°C in a *daf-12*/NHR-dependent manner [2]. Together these findings suggest that *daf-10* mutants tend to become dauers at high temperature because they have basally altered activity of DAF-12/NHR, as well as DAF-16/FOXO.

### Sensory mutations lead to down-regulation of pathogen-response genes

**Figure 3:**
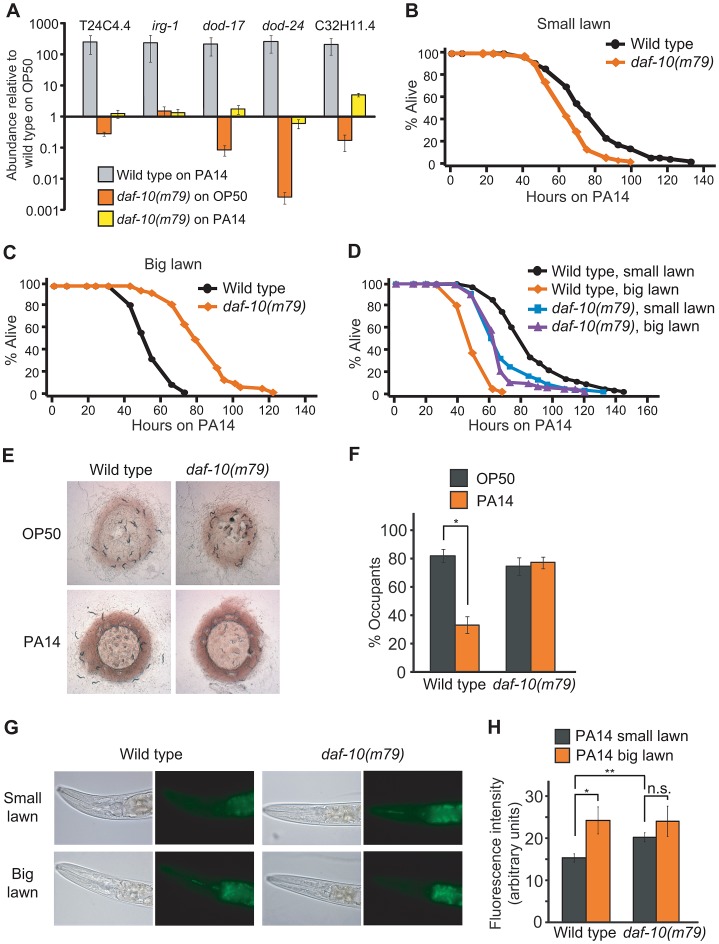
*daf-10* mutations alter the worm's response to pathogens. A. qRT-PCR analysis was performed to determine how five pathogen (PA14)-responsive genes were regulated in *daf-10(m79)* mutants in the presence of *E. coli* OP50, the normal laboratory food, or of pathogenic *P. aeruginosa* PA14 (big lawn). B–D. *daf-10(m79)* animals showed opposite phenotypes when fed *P. aeruginosa* in a small lawn assay (B) or in a big lawn assay (C). When the two assays were done in parallel (D), the survival of *daf-10(m79)* animals was similar in both conditions. In contrast, the survival of wild-type animals was dramatically reduced when a big lawn was used. A summary of the data presented in these panels and additional repeats is included in Table S2. E–F. Whereas wild-type animals avoided PA14, *daf-10(m79)* animals occupied lawns of *E. coli* OP50 and *P. aeruginosa* PA14 bacteria to a similar degree. Approximately 100 wild-type and *daf-10(m79)* worms were placed on lawns of PA14 and OP50 and imaged after 16 hrs (E). The % of worms that occupied (% occupants) the bacterial lawn was also determined (F). G–H. Representative pictures of the fluorescence signal from GFP-labeled PA14 bacteria in the intestine of worms (G) and quantitation of fluorescence signal (n≥26 worms per strain/condition) (H), showing the amount of PA14 ingested in small vs. big lawn assays by wild-type and *daf-10(m79)* worms. Error bars represent s.e.m. (^n.s.^ *p*>0.05, * *p*<0.05, ** *p*<0.01, Student's *t-*test).

Because some of the genes in our dataset were annotated as pathogen-responsive genes, we compared the genes differentially expressed in sensory mutants with the transcriptional profiles of *C. elegans* exposed to the bacterial pathogen *P. aeruginosa* PA14 [32], [33]. We found that a significant number of the genes down-regulated in *daf-10* mutants on normal *E. coli* OP50 diet were up-regulated in response to exposure to PA14 in adult wild-type animals (Out of 56 down-regulated genes, 15 were classified as up-regulated by PA14 exposure in Shapira et al. [32] and 21 in Troemel et al. [33], *p*<0.0001 in both cases, hypergeometric probability, Table 3). Using quantitative RT-PCR analysis, we confirmed that four out of five PA14-responsive genes selected from our microarray analysis were down-regulated in *daf-10* mutants on an OP50 diet (Figure 3A). In addition, we also found that *daf-10* mutations resulted in a severe reduction in the induction of four out of the five genes following PA14 exposure (Figure 3A). These data suggest that the sensory system is required not only for the basal expression of these pathogen-responsive genes when animals are fed the normal laboratory food, OP50, but also for their full induction upon PA14 pathogen exposure. We then tested whether the *daf-10* sensory mutants had an altered response to pathogens by measuring their survival on *P. aeruginosa*. Under standard testing conditions, we found results to be highly variable, but could detect a significant decrease in the survival of *daf-10(m79)* animals on PA14 in four out of ten trials (Figure 3B, Table S2).

Interestingly, a seemingly unrelated sensory system mutant, the *npr-1* mutant, which has a defect in sensing oxygen concentrations, also presents an increased sensitivity to pathogens [9], [11]. Indeed, the shorter survival of *daf-10(m79)* mutant animals on *P. aeruginosa*, like that of *npr-1* mutant animals [11], was dependent on the oxygen-sensing guanylate cyclase *gcy-35*, because *gcy-35(ok769); daf-10(m79)* animals were as resistant to *P. aeruginosa* as *gcy-35(ok769)* animals (Figure S2A). Although *npr-1* mutant animals are more sensitive than wild-type animals under the usual “small lawn” assay conditions, their survival is similar to that of wild-type animals if assayed on a plate completely covered with bacteria (big lawn) [9]. This is likely because wild-type animals actively avoid the pathogenic bacteria in small lawn assays, but cannot do so in big lawn assays, whereas *npr-1* mutants actively seek the lower oxygen concentrations provided by the thick *P. aeruginosa* bacterial lawn even in the small lawn assay [9]. When we used a big lawn to test the survival of *daf-10(m79)* animals on PA14, we found that not only *daf-10(m79)* animals were not short lived under these conditions, but they were in fact longer lived than wild-type animals (Figure 3C). This may indicate that *daf-10(m79)* animals are physiologically more resistant to pathogens, but are behaviorally unable to avoid them. When the assays were done in parallel, the survival of wild-type animals was 40% shorter in the big lawn assay than in the small lawn assay, whereas the survival of *daf-10* mutants was not significantly different between the two conditions (Figure 3D). Consistent with these data, wild-type animals avoided PA14 and had lower occupancy of the PA14 bacterial lawn compared to the OP50 lawn, whereas *daf-10* mutants displayed defects in avoiding PA14 (Figure 3E and 3F and Figure S2B). In addition, *daf-10(m79)* mutants ingested more GFP-PA14 than wild-type animals in the small lawn assay (Figure 3G and 3H).

**Figure 4:**
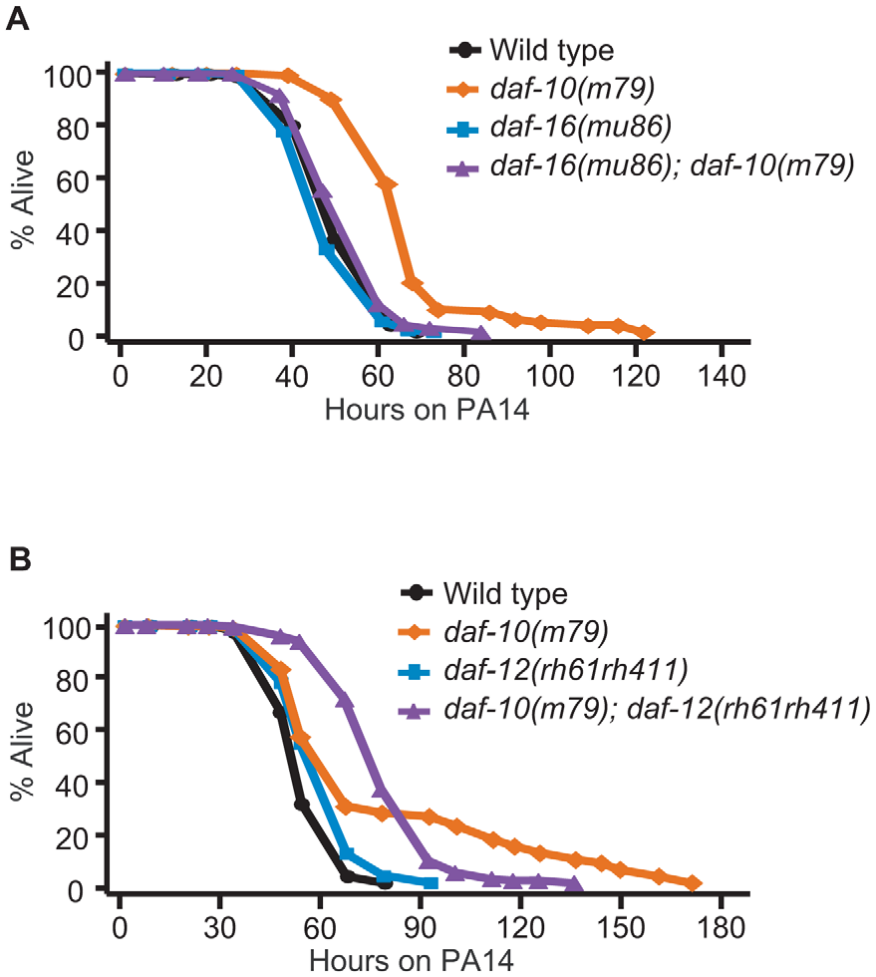
The increased PA14 resistance of *daf-10* mutants requires DAF-16/FOXO. *daf-16(mu86)* mutations completely suppressed the PA14 resistance caused by *daf-10(m79)* mutations in the big lawn assay (A), whereas *daf-12(rh61rh411)* mutations did not affect mean survival (B). However, *daf-12* mutations altered the survival curve of *daf-10(m79)* mutants on PA14 in a statistically significant manner (see Figure S3 for additional repeats). A summary of the data presented in these panels and additional repeats is included in Table S2.

Increased DAF-16/FOXO confers resistance to various pathogenic bacteria including PA14 [14], and we found that DAF-16/FOXO was required for the pathogen resistance of *daf-10(m79)* animals in the big lawn assay (Figure 4A). In contrast, *daf-12*/NHR mutation did not significantly affect the average survival time of *daf-10* mutants on PA14 (Figure 4B). However, *daf-12* mutations shortened the maximal survival time of *daf-10(m79)* animals on PA14, while increasing their survival at early time points. The difference in the survival curves was significant in three out of five trials when using the Wilcoxon test, which does not assume constant hazard ratios [40] (Figure S3 and Table S2). This may indicate a more subtle and complex effect of DAF-12/NHR on the response to pathogens, perhaps aiding in long-term survival but negatively impacting responses after short exposures. Alternatively, loss of *daf-12* could simultaneously affect the activity of two different neuronal populations with opposing roles in regulating sensitivity to PA14, and thus result in a complex phenotype.

We have thus shown that *daf-10(m79)* animals have decreased expression of pathogen-responsive genes even when exposed to *E. coli*, but are in fact physiologically more resistant to pathogenic bacteria such as *P. aeruginosa* due to increased DAF-16/FOXO activity. In contrast, behavioral avoidance of *P. aeruginosa* is impaired in *daf-10(m79)* mutant worms, underscoring the importance of sensory input in the behavioral response to pathogens.

### The up-regulated gene *mct-1/2* is required for the lifespan increase of *daf-10(m79)* sensory mutant animals

**Figure 5:**
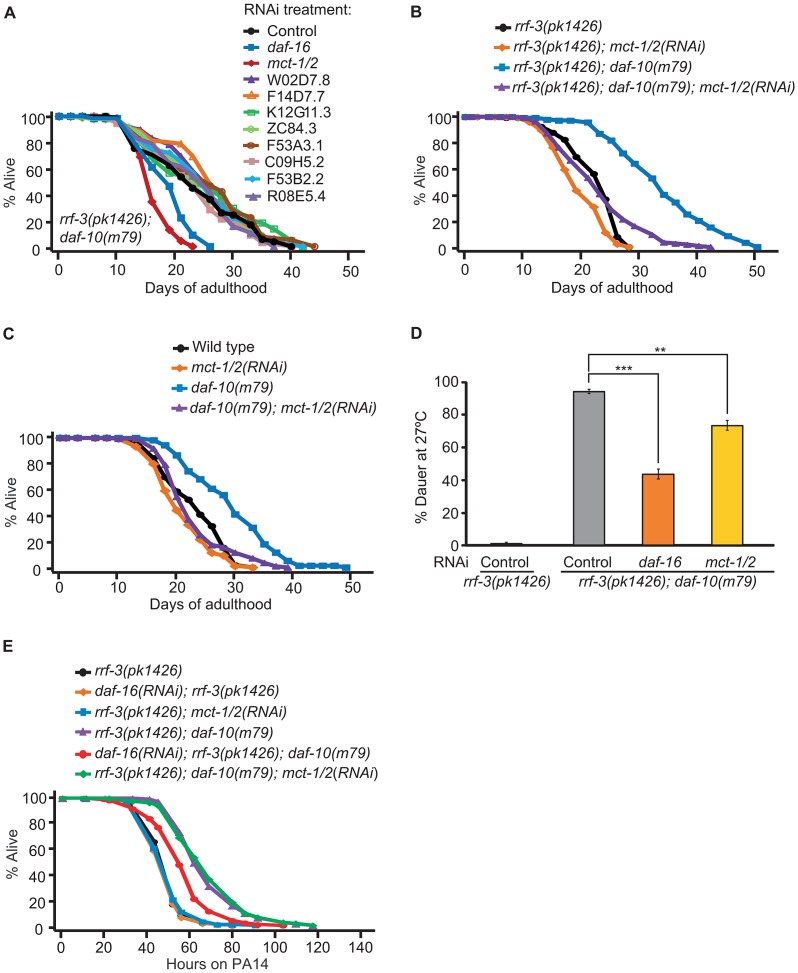
*mct-1/2* is required for the long lifespan and dauer formation of *daf-10* mutants. A. RNAi bacteria for 9 of the 14 up-regulated genes were obtained from the *C. elegans* RNAi libraries [65], [66]. *rrf-3(pk1426); daf-10(m79)* animals were treated with RNAi targeting the nine genes, control RNAi (containing empty vector) or *daf-16* RNAi bacteria. The latter, as shown previously [3], shortened lifespan of the sensory mutant animals and served as a positive control. RNAi against *mct-1/2* significantly decreased the long lifespan of *rrf-3(pk1426); daf-10(m79)* animals. B. RNAi targeting *mct-1/2* almost completely suppressed the longevity of *rrf-3(pk1426); daf-10(m79)* animals, while having little effect on that of *rrf-3(pk1426)* animals. C. *mct-1/2* was knocked down by RNAi in wild-type and *daf-10(m79)* animals. When grown on *mct-1/2* RNAi, the life-extending effect of *daf-10(m79)* mutations was significantly reduced. D. *mct-1/2* RNAi partially but significantly suppressed the constitutive dauer formation phenotype of *daf-10(m79)* mutants at 27°C. *daf-16* RNAi was used as a positive control. Error bars represent s.e.m. (** *p*<0.01, *** *p*<0.001, Student's *t-*test). E. *mct-1/2* RNAi did not affect the PA14 resistance due to *daf-10(m79)* mutations in the big lawn assay, whereas *daf-16* RNAi did. The RNAi-hypersensitive *rrf-3(pk1426)* mutant background was used to potentiate the RNAi effect. A summary of the data presented in this figure is included in Tables S1B and S2.

Genes that are up-regulated in response to sensory system mutations may also be required for their effects on physiology. We focused on the role of these genes in lifespan regulation and tested whether knocking them down by RNA interference (RNAi) affected the lifespan of *rrf-3(pk1426); daf-10(m79)* animals. As neurons are usually refractory to RNAi, the *rrf-3* mutation was used to increase neuronal sensitivity to the treatment [41], [42]. We were able to test 9 of the 14 up-regulated genes (for the remaining 5, RNAi clones were not available or did not grow). We identified one that was required for the extended longevity of *rrf-3(pk1426); daf-10(m79)* animals, the uncharacterized gene Y19D10A.12, which we named *mct-1* since it encodes a putative monocarboxylate transporter (Figure 5A and 5B). *mct-1* is located within a region of chromosome V that has been duplicated [43]. Therefore, chromosome V contains a second gene, C01B4.9 or *mct-2*, which is almost identical to *mct-1* (Figure S4). Based on the reference sequences and gene models available in Wormbase WS230, *mct-2* has a 319 base pair (bp) insertion in the intronic region between exon 2 and exon 3. In addition, the splicing of the 3rd exon of *mct-1* and *mct-2* differs, so that the *mct-2* 3rd exon likely contains 81 additional nucleotides at its 3′ end (Figure S4). The promoter sequence of the two genes also appears to be almost identical, the only difference being a single guanosine insertion in a G stretch 1629 bp 5′ of the start codon in *mct-1* (Figure S4). This suggests that the expression and regulation of *mct-1* and *mct-2* is likely to be very similar. Because the RNAi treatment is likely to affect both genes and our qPCR primers do not distinguish between them (Figure S4), we are unable to separate their actions and we will henceforth refer to these genes collectively as *mct-1/2*.

Given our initial finding, we decided to further characterize the effects of *mct-1/2* and found that *mct-1/2* knockdown also shortened the long lifespan of *rrf-3(+); daf-10(m79)* strains, while wild-type animals were largely unaffected (Figure 5C). *mct-1/2* was also required for the increased dauer formation of *daf-10(m79)* animals at high temperature (Figure 5D). In contrast, knockdown of *mct-1/2* did not affect the increased PA14 resistance of *daf-10(m79)* animals in the big lawn assay (Figure 5E).

**Figure 6:**
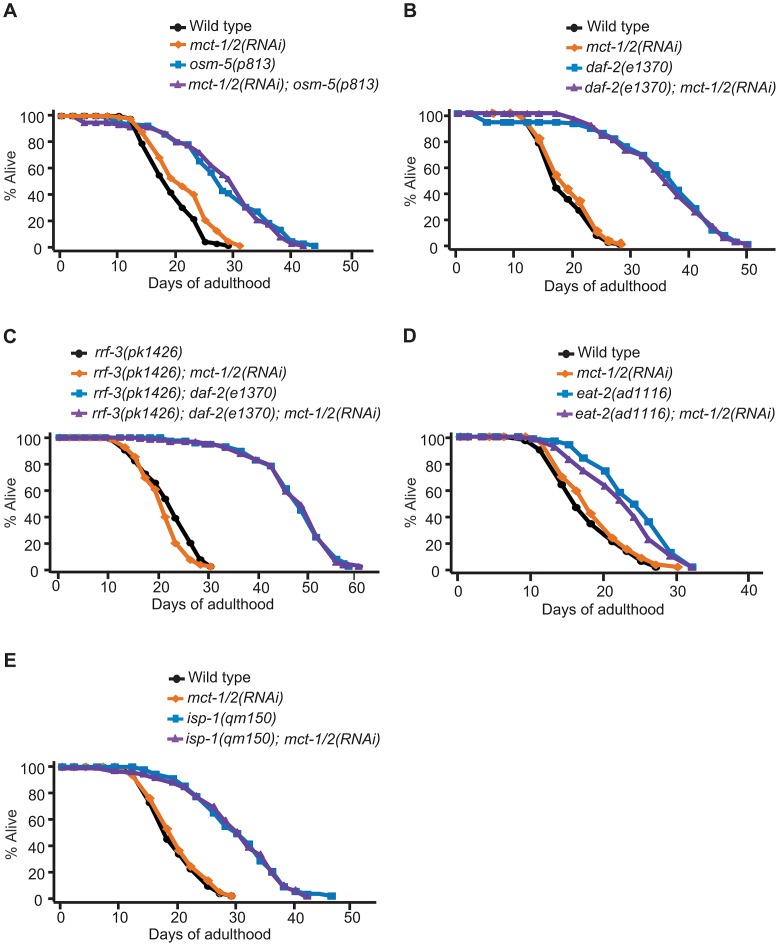
*mct-1/2* is dispensable for the longevity of various long-lived mutants. Reduction of *mct-1/2* levels by RNAi did not shorten the lifespan of *osm-5(p813)* sensory mutants (3 out of 4 times) (A), *daf-2(e1370)* mutants in either *rrf-3(+)* (B) or *rrf-3(−)* (C) backgrounds, dietary-restricted *eat-2(ad1116)* animals (D) or mitochondrial respiration-impaired *isp-1(qm150)* mutants (E). A summary of the data presented in this figure and additional repeats is included in Table S1B.

To determine the role of *mct-1/2* in other lifespan-extending pathways, we tested whether *mct-1/2* RNAi could influence the lifespan of other long-lived *C. elegans* mutants. We found that the longevity of another long-lived sensory mutant strain, *osm-5(p813)*, was not influenced by reduction in *mct-1/2* levels (Figure 6A). This is consistent with our quantitative RT-PCR results showing that the mRNA levels of *mct-1/2* were increased in *daf-10* mutants but not in *osm-5* mutants (Figure 1B and Figure S1B). Moreover, it suggests previously unappreciated differences in longevity regulation between these two sensory mutants. We also observed that RNAi knock-down of *mct-1/2* had little or no effect on the long lifespan of *daf-2*/InsR mutants in either *rrf-3(+)* or *rrf-3(−)* backgrounds (Figure 6B and 6C), despite the fact that both *daf-2*/InsR and *daf-10* mutant animals require DAF-16/FOXO for their longevity. Lastly, the extended longevity of dietary-restricted *eat-2* mutants or mitochondrial respiration-defective *isp-1* mutants was largely unaffected by *mct-1/2* RNAi treatment (Figure 6D and 6E). These data suggest that the requirement of *mct-1/2* for longevity regulation exhibits specificity for *daf-10* sensory mutants.

**Figure 7:**
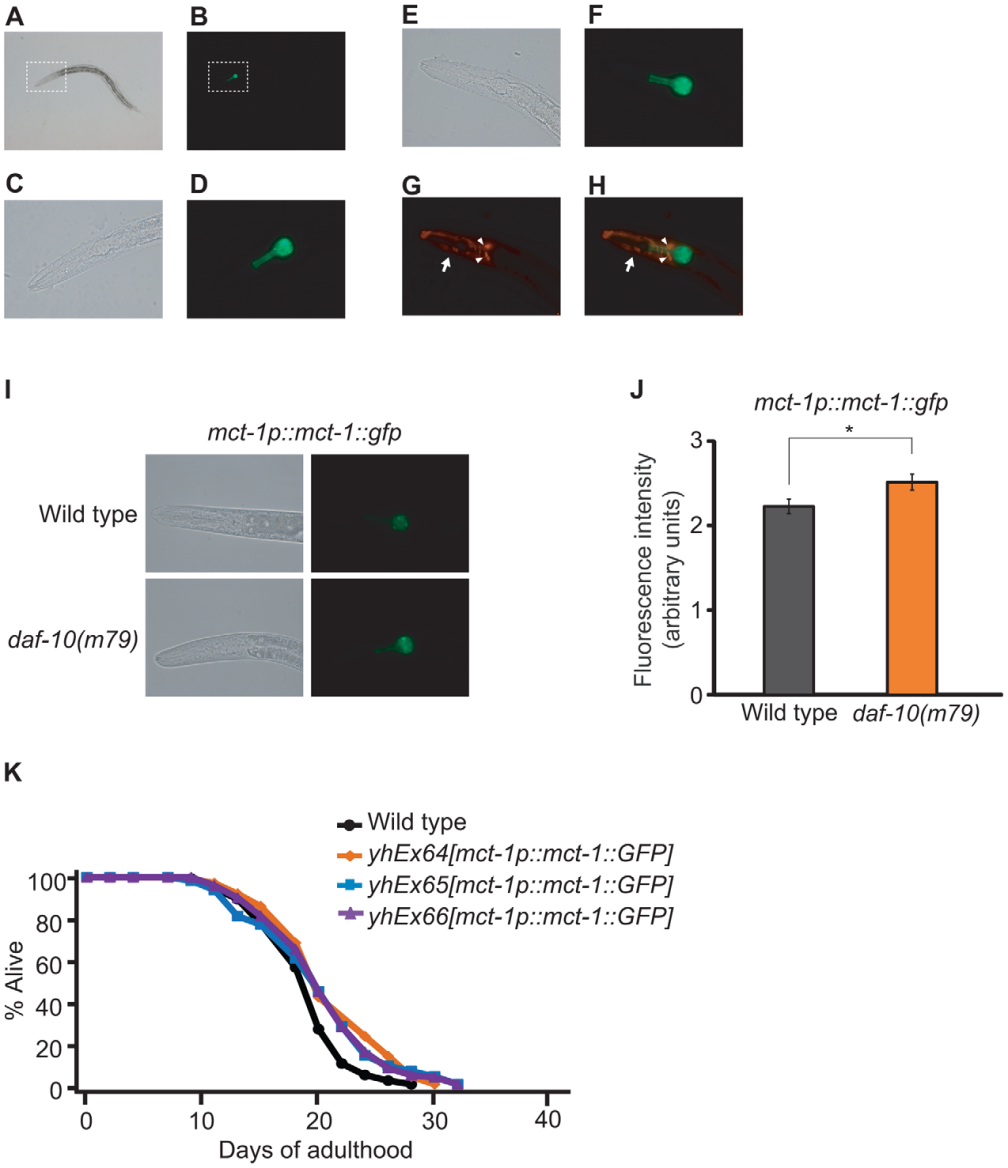
Overexpression of *mct-1* extends lifespan. A–D. Expression pattern of GFP-fused MCT-1 protein in *mct-1p::mct-1::GFP* transgenic animals. Bright field image (A) and green fluorescence image (B) of an L4 *mct-1p::mct-1::GFP* transgenic larva. Magnified bright field image (C) and green fluorescence image (D) of dotted box regions in A and B respectively. E–H. The expression of GFP-fused MCT-1 protein in the *mct-1p::mct-1::GFP* transgenic animal did not overlap with the signal from amphid sensory neurons labeled with the DiI dye. Bright field image (E), green fluorescence image (F), red fluorescence image (G) and overlay of green and red fluorescence images (H) of the head of an L3 *mct-1p::mct-1::GFP* transgenic animal. Arrowheads indicate cell bodies of amphid sensory neurons that are stained with DiI dye. The arrow indicates body wall muscle that expresses a muscle-specific *myo-3p::RFP* transgene, used as a marker for the generation of the transgenic animals. I–J. Expression pattern of GFP-fused MCT-1 protein in *daf-10(m79)* mutants, including representative green fluorescence and bright field images (I) and quantification of fluorescence intensity (J). Error bars represent s.e.m. (* *p*<0.05, Student's *t-*test). K. Overexpression of *mct-1::GFP* lengthens lifespan. Three independent lines of *mct-1* transgenic animals lived slightly but significantly longer than control animals. Statistical analysis of the lifespan data presented in this figure and additional repeats is included in Table S1B.

We decided to examine the expression pattern of *mct-1/2* and to test whether *mct-1/2* overexpression was sufficient to extend the lifespan of otherwise wild-type worms. We designed primers that would amplify the promoter and the cDNA of both genes, and we were able to clone promoter and cDNA sequences matching the gene model for *mct-1*. We then generated transgenic animals that express GFP-tagged MCT-1 protein under the *mct-1* promoter (*mct-1p::mct-1::GFP*) and found that *mct-1* was expressed in the pharynx (Figure 7A–7D). The expression pattern persisted from hatching to adulthood (data not shown), and GFP-tagged MCT-1 was not detected in six amphid sensory neurons that are labeled by DiI dye (Figure 7E–7H). As expected from the microarray and quantitative RT-PCR data, we found that *daf-10(m79)* mutations increased expression of the *mct-1p::mct-1::GFP* transgene in the pharynx, whereas the expression pattern of MCT-1::GFP was not altered by *daf-10(m79)* mutations (Figure 7I and 7J). These data imply that the sensory neuronal defects of the *daf-10* mutants may increase the expression of *mct-1* in the pharynx to promote longevity and dauer formation. In agreement with this hypothesis, overexpression of the MCT-1 transporter slightly but significantly extended the lifespan of otherwise normal animals in two different trials using three independent transgenic lines (Figure 7K and Table S1B). Collectively, these data indicate that a previously uncharacterized gene, *mct-1/2* is specifically induced by certain sensory system alterations and contributes to the longevity associated with sensory system defects.

## Discussion

In this study, we sought to understand how changes in transcriptional programs downstream of alterations in sensory perception may lead to changes in organismal physiology such as extended longevity and altered developmental programs. We examined the whole-organism transcriptional profile of worms carrying a mutation in a gene encoding the worm homolog of the intraflagellar transport protein IFT222, *daf-10*. *daf-10* mutations cause broad defects in the sensory system of the worms. As expected based on previous genetic data, the expression of targets of the transcription factor DAF-16/FOXO was altered in *daf-10(m79)* animals, confirming the validity of our approach. Moreover, we showed that the activity of another transcription factor involved in the regulation of lifespan and dauer formation, DAF-12/NHR, was also affected in *daf-10* mutant worms and that DAF-12/NHR was specifically required for dauer formation. We also found that one of the genes that were up-regulated in *daf-10(m79)* animals, the monocarboxylate transporter homolog *mct-1/2*, was required for the longevity of sensory mutant animals. Lastly, we uncovered a high representation of immune response genes among the genes whose expression was reduced by the sensory mutation, which was likely related to the reduced pathogen avoidance of sensory mutant animals.

The up-regulation of DAF-16/FOXO targets in *daf-10(m79)* mutants is in accord with the fact that *daf-*16/FOXO is largely required for their longevity and their increased dauer formation. Our data further support the idea that one or multiple insulin-like peptides are misregulated in response to sensory system mutations contributing to the lifespan increase of worms with defective sensory neurons. It is interesting, however, that some genes that are typically used as reporters of DAF-16/FOXO activity, such as *sod-3* and *mtl-1*, were not up-regulated in *daf-10(m79)* mutants based on our microarray data. qPCR analysis confirmed this observation (Figure S5). This hints at an unappreciated complexity in the relationship between the sensory system and insulin/IGF-1 signaling in *C. elegans*.

Our microarray data also support regulation of DAF-12/NHR activity by the sensory system because of the reduced expression of DAF-12/NHR target genes in *daf-10(m79)* mutant animals. This interpretation is consistent with the observation that *daf-*12/NHR is required for the increased dauer formation at high temperatures of the *daf-10* (Figure 2E) and *osm-5* sensory mutants (data not shown), as well as the *tax-4* sensory mutant as previously reported [2]. In fact, *daf-12*/NHR is necessary for dauer formation under a range of conditions, which suggests either that many different dauer-inducing conditions affect the activity of DAF-12/NHR, or that basal expression of DAF-12/NHR target genes is required for entry in the dauer state. Because we observe that the DAF-12/NHR target genes are mostly unchanged in *daf-12(rh61rh411)* mutant animals relative to wild-type animals, we conclude that the first possibility is more likely, at least as far as sensory mutant worms are concerned, and that the sensory system actively regulates the *daf-12*/NHR signaling pathway.

The fact that lifespan, dauer formation and immune response (see below) are whole-organism events implies that hormonal signaling downstream of the sensory system may be necessary to coordinate whole-body changes. Whereas steroid signals through DAF-12/NHR may fulfill that role for dauer formation, it is unclear at present what such a signal may be for the lifespan phenotype. Among the genes that were up-regulated in *daf-10(m79)* animals, one gene, *mct-1/2*, was also required for the extended longevity of these mutants. *mct-1* and its nearly identical paralog, *mct-2*, are homologous to monocarboxylate transporters, which can transport small molecule hormones such as thyroid hormones in mammalian systems [44]. Moreover, expression of a *Drosophila melanogaster* homolog of monocarboxylate transporters is altered in flies after glucose feeding [45], suggesting a potential link to insulin/IGF-1-like signaling. Thus, it is possible that the specific role of *mct-1/2* in the lifespan of sensory mutant animals is linked to the signals that help coordinate the development and aging of different tissues. In addition, *mct-1/2* is one of the first genes to be identified as a specific regulator of longevity downstream of the sensory system. Further analysis of the biochemical function of this gene will thus provide crucial information on how sensory perception regulates lifespan. Our finding that *mct-1/2* is not required for the longevity of *osm-5* mutant animals suggests that *daf-10* and *osm-5* sensory mutations control divergent downstream pathways, rather than converging on the same regulatory mechanism. This is consistent with the differential effect these mutations have on subsets of sensory neurons [18], despite the fact that they both cause pervasive defects in the development of amphid sensory cilia. For example, the cilia of some amphid neurons (ADF, ASH, ALD) are still partially intact in *daf-10(m79)* mutants, and these animals are not defective in chemotaxis toward NaCl, unlike *osm-5* animals [18]. Conversely, *daf-10(m79)* animals are defective in male mating, whereas *osm-5(p813)* animals are not, suggesting the *daf-10* mutation leads to stronger impairment of neurons involved in mating [18]. This is also consistent with previous studies showing that multiple sensory neurons control different pathways that regulate lifespan, and can influence longevity in both a positive and a negative direction [3], [6], [8]. Moreover, we cannot exclude that the mutations have different effects on signaling even in those neurons where the morphological effects are similar.

The microarray data also point at a role for the neurons affected by *daf-10(m79)* mutations in regulating the response to bacterial pathogens such as *Pseudomonas aeruginosa*. *daf-10* mutations not only alter the expression of genes that are normally responsive to *P. aeruginosa* exposure, but also prevent worms from leaving the lawn of pathogenic bacteria and simultaneously increase the resistance of the animals to *P. aeruginosa*. In recent studies, several different components of the sensory system, including oxygen-sensing neurons [9], [11], serotonin pathways [10], [13] and neuropeptide pathways [46], have been implicated in the two main protective responses to *P. aeruginosa* exposure: up-regulation of protective anti-microbial genes and decreased pathogen consumption due to avoidance of the bacterial lawn. Because the *daf-10* mutations affect the function of a number of ciliated neurons, it is unclear at present how different sensory modalities may contribute to the phenotype of *daf-10(m79)* animals. For example, the oxygen-sensing *gcy-35*-expressing neurons AQR and PQR [47], [48] are ciliated neurons, and the *daf-10* survival phenotype on small PA14 lawns resembles that of the *npr-1* mutant, in which these neurons are hyperactive [49]. It is possible that in *daf-10(m79)* animals, lack of proper sensory cilia in AQR and PQR results in hyperactive signaling from the neurons, preventing the animals from leaving the lawn. The subsequent increased pathogen consumption would then mask the physiological resistance to pathogens that becomes apparent in the big lawn assay. This interpretation would be consistent with the fact that mutations in the oxygen-sensing guanylate cyclase *gcy-35* suppress the increased pathogen sensitivity of *daf-10(m79)* mutants in the small lawn assay (Figure S2A). Hyperactivation of defective sensory neurons is also consistent with the paradoxical observation that *daf-10* mutants are considered Daf-d (dauer defective) because they are insensitive to dauer pheromone [50], and yet these animal arrest more readily as dauers at high temperature [2], [3]. In contrast, the decreased sensitivity of *daf-10(m79)* animals to PA14 in the big lawn assay likely results from the activation of the DAF-16/FOXO transcription factor. Although this “competing signals” model is consistent with the data, we also noticed that the survival of *daf-10(m79)* animals was similar between the two types of PA14 resistance assays, while the survival of wild-type animals was shorter in the big lawn assay. This suggests an alternative model whereby reduced pathogen perception due to sensory mutations results in the inability of *daf-10(m79)* mutant animals to modulate their physiology and their behavior in the presence of different bacterial strains. The altered expression of pathogen response genes may also stem from an inability of *daf-10(m79)* animals to perceive the presence of *Escherichia coli*, which may be slightly pathogenic for worms [51], [52].

The importance of the neuronal regulation of many basic physiological processes has been appreciated only recently. In mammals, hormonal release regulated by the hypothalamus has an important role in energy homeostasis, fat storage, immune responses and possibly aging (reviewed in [53]). For example, recent studies have shown that loss of the IGF-1 receptor specifically in the brain has profound effects on energy homeostasis and results in increased lifespan by changing the growth hormone axis [54]. Interestingly, the hypothalamus receives direct innervation from many cortical areas devoted to sensory processing [55], including the olfactory cortex and the insular cortex, which processes gustatory information, pointing at a clear role for environmental input in the regulation of physiology. Although the *C. elegans* circuitry is much more compact than that of mammals, studying the signal relay from the nervous system to peripheral tissues in this simple, genetically tractable organism, may give insights into what pathways may be used in higher organisms to mediate similar signals. In this context, our study highlights several transcriptional changes that correspond to precise phenotypes, providing examples of how neuronal signaling changes may be translated into an organismal physiological output.

## Materials and Methods

### Strains

Nematodes were raised under standard laboratory conditions on agar plates containing a lawn of *Escherichia coli* strain OP50, as described previously [56]. “Wild type” was the *C. elegans* strain N2. The mutant and transgenic strains used were as follows: CF2100 *daf-10(m79)*, CF2479 *daf-12(rh61rh411)*, CF2553 *osm-5(p813)*, CF3152 *rrf-3(pk1426)*, CF3295 *daf-10(m79); daf-12(rh61rh411)*, CF3302 *daf-16(mu86); daf-10(m79)*, CF3369 *daf-16(mu86); daf-10(m79); daf-12(rh61rh411)*, CF3389 *gcy-35(ok769)*, CF3390 *gcy-35(ok769); daf-10(m79)*, CF3391 *rrf-3(pk1426); daf-10(m79)*, CF1814 *rrf-3(pk1426); daf-2(e1370)*, CF1041 *daf-2(e1370)*, CF2172 *isp-1(qm150)*, CF1908 *eat-2(ad1116)*, IJ294 *yhEx64[mct-1p::mct-1::gfp, myo-3p::rfp]*, IJ295 *yhEx65[mct-1p::mct-1::gfp, myo-3p::rfp]*, IJ296 *yhEx66 [mct-1p::mct-1::gfp, myo-3p::rfp]*, IJ374 *daf-10(m79); yhEx64 [mct-1p::mct-1cDNA::gfp, myo-3p::rfp]*. All strains were outcrossed to an isogenic N2 strain at least three times.

### Microarray analysis

To make sure that background differences did not affect our results, prior to doing microarray analysis we outcrossed *daf-10(m79)* animals four times to our N2 strain. We confirmed that the outcrossed *daf-10(m79)* animals lived approximately 30% longer than wild-type animals and that this increase was largely dependent on *daf-16*/FOXO (data not shown). Wild-type (N2) and *daf-10(m79)* worms were synchronized by arresting at L1 overnight in M9 buffer. They were then grown at 20°C and collected as young adults. Total RNA was purified using TriZol reagent (Invitrogen), and mRNA was purified using Oligotex kit (Qiagen). cDNA was generated, coupled to Cy3/Cy5 dyes and hybridized to single-stranded DNA nucleotide arrays printed in-house using standard techniques. The oligonucleotides were purchased from Illumina and represented 20,374 unique *C. elegans* genes. Three repeats of a direct comparison between wild-type and *daf-10(m79)* animals were carried out. Chips were scanned using a GenePix 4000B scanner, and initial quality check and identification of spots was done using Genepix 6.0 software. Linear normalization was carried out with the Acuity 4.0 software and significance analysis using the Cyber T-test program [57]. Genes with a *p* value lower than 0.02 were considered significant. The significant gene list was compared to known gene lists using the hypergeometric probability. Microarray data have been deposited in the Gene Expression Omnibus database under accession number GSE41943.

### Quantitative RT–PCR

RNA extraction using TriZol reagent (Invitrogen), purification using RNeasy kit (Qiagen) and reverse transcription using Proto-Script 1^st^ strand cDNA synthesis kit (NEB) were performed as described [58]. Quantitative RT-PCR was performed using a 7300 Real Time PCR System (Applied Biosystems) and analyzed by the Ct method (Applied Biosystems Prism 7700 Users Bulletin No. 2 http://docs.appliedbiosystems.com/pebiodocs/04303859.pdf). mRNA levels of *act-1*, *nhr-23* and *ama-1* were used for normalization. Raw values were normalized to each of the three control genes and the average of the normalized values was used as the data point. The average of at least two technical repeats was used for each biological repeat. The data shown in the figures are the average of at least three biological repeats. Primer sequences are listed in Table S3.

### Lifespan analysis

Lifespan analysis was conducted as described previously [59]. All assays were carried out at 20°C. For RNAi experiments, eggs were placed on lawn of double-stranded RNA-expressing *E. coli* HT115. The lifespan of the progeny of these animals was assayed on RNAi bacteria. A bacterial strain containing an empty vector was used as control. Some assays were carried out in the presence of 75 µM 5′-fluorodeoxyuridine (Sigma). OASIS (online application for the survival analysis, http://sbi.postech.ac.kr/oasis) was used to analyze the data [60], and *p* values were calculated using the log-rank test (Mantel-Cox method).

### Pathogen resistance analysis

Pathogen resistance assays were carried out as described with minor modifications [61]. *Pseudomonas aeruginosa* strain PA14-coated plates were made by seeding 5 µl of an overnight culture of PA14 in Terrific broth and incubating at 37°C overnight. For big lawn assays, 15 µl of cultured PA14 overnight was seeded and spread in order to cover the whole surface of the plate, and incubated 37°C overnight [9]. Plates were subsequently kept in a 25°C incubator. Worms were grown on *E. coli* OP50 and placed on PA14 plates at day 1 of adulthood. Plates were examined one to three times per day, and animals were scored as alive if they moved when prodded. Animals that crawled onto the sides of the plates (where they died of desiccation) were excluded from the analysis. OASIS (Online Application for the Survival Analysis, http://sbi.postech.ac.kr/oasis) [60] was used to analyze the data, and *p* values were calculated using the log-rank test (Mantel-Cox method) and the Wilcoxon test, which does not assume constant hazard ratios [40], [62], because differences in the shape of the survival curves for one set of data [*daf-10(m79)* vs. *daf-10(m79); daf-12(rh61rh411)*] suggested the latter test may be more appropriate for some of the comparisons.

### GFP-PA14 intake assays

The assays were performed as described [9] using GFP-expressing *Pseudomonas aeruginosa* (GFP-PA14) to measure PA14 intake. Worms were grown on OP50 until they reached adulthood, and then placed on plates seeded with small and big lawns of GFP-PA14 for 24 hours. Images of GFP-PA14-fed worms were captured using AxioCam (Zeiss Corporation, Germany) with HRc Zeiss Axioscope A.1 (Zeiss Corporation, Germany). GFP fluorescence was quantified using ImageJ (http://rsbweb.nih.gov/ij/).

### Pathogen avoidance assays

The assays were performed as described with minor modifications [63]. Five µl of cultured PA14 or OP50 was seeded on high peptone (0.35%) agar plates to prepare plates with small lawns of bacteria. After incubation for 24 hours at 37°C, plates were moved to 25°C and used for the avoidance assay within 24 hours. Approximately 100 young (day 1) adult worms were transferred onto plates seeded with PA14 or OP50. The number of occupants was counted every 4 hours for 16 hours after the transfer. Images of the plates were captured using DIMIS-M (Siwon Optical Technology, South Korea) camera.

### Dauer assays

Embryos were incubated at 27°C for 2 days, and plates were then examined for the presence of dauer larvae. For the RNAi experiment in Figure 5D, *rrf-3(pk1426); daf-10(m79)* mutant embryos were placed on plates seeded with control bacteria (containing empty vector), or bacteria expressing double-stranded RNA targeting *daf-16*/FOXO or *mct-1/2* at 20°C. The progeny of these animals were transferred to 27°C as embryos, and scored for the presence of dauer larvae two days later. The experiments were repeated eight times.

### Generation and analysis of transgenic animals

To generate transgenic animals overexpressing GFP-fused MCT-1, approximately 2 kb upstream of the *mct-1* coding region and the cDNA of *mct-1* were PCR amplified. The PCR products were then fused to Gateway donor vectors using BP clonase (Invitrogen) followed by recombination with a Gateway destination vector containing *GFP* and *unc-54* 3′UTR using LR clonase (Invitrogen). The DNA construct was injected into the gonads of young adults at a concentration of 25 ng/µl with 75 ng/µl of a *myo-3p::RFP* marker. Images of transgenic worms were captured using AxioCam (Zeiss Corporation, Germany) with HRc Zeiss Axioscope A.1 (Zeiss Corporation, Germany). GFP fluorescence was quantified using ImageJ (http://rsbweb.nih.gov/ij/).

### Di<$>\scale 130%\raster="rg1"\<$> staining

DiI staining was performed as described previously with some modifications [64]. DiI staining solution was prepared by diluting DiI (1,1′-dioctadecyl-3,3,3′,3′-tetramethylindocarbocyanine perchlorate, Invitrogen) in M9 buffer to a final concentration of 40 µg/ml. Approximately 30 L4 larvae were washed with M9 buffer three times and incubated in the DiI staining solution for 2 hours at 20°C while shaking (200 rpm). The worms were then washed with M9 buffer three times and placed on OP50-seeded NGM plates for 10 minutes. They were subsequently mounted on 2% agarose pads and anesthetized with 100 mM sodium azide. Images of the stained worms were captured using an AxioCam HRc (Zeiss Corporation, Germany) camera attached to a Zeiss Axioscope A.1 microscope (Zeiss Corporation, Germany).

## Supporting Information

Figure S1 *osm-5(p813)* mutations affect the expression of some, but not all, genes differentially regulated in *daf-10(m79)* animals. A–B. qRT-PCR was used to examine how the expression of genes that were down-regulated (A) or up-regulated (B) in *daf-10(m79)* mutant animals based on the microarray analysis was altered in another chemosensory mutant, *osm-5(p813)*. The general trend was that ∼50% of the genes were also differentially regulated in *osm-5(p813)* animals. However, perhaps due to large variability between biological repeats, not all data sets were statistically significant. Error bars represent s.e.m. * *p*<0.05, ** *p*<0.01, the Student's *t-*test.
(EPS)

Figure S2 In the small lawn assay, *daf-10* mutants require the oxygen-sensing guanylate cyclase *gcy-35* for decreased survival and display defects in avoiding PA14. A. The oxygen-sensing guanylate cyclase *gcy-35* is required for the decreased survival of *daf-10(m79)* animals in the PA14 small lawn assay. *gcy-35(ok769)* mutations prevented *daf-10(m79)* animals from living shorter than wild type when cultured on small lawns of *P. aeruginosa* PA14. In addition, the *gcy-35* mutation alone conferred slight resistance to *P. aeruginosa* in this assay. A summary of the data presented here is included in Table S2. B. The % of wild-type and *daf-10(m79)* mutant worms that occupied (% occupants) the bacterial lawn was determined at different time points after the transfer from *E. coli* OP50 plates. Error bars indicate s.e.m. from three independent experiments.
(EPS)

Figure S3 Survival curves of wild-type, *daf-10(m79)*, *daf-12(rh61rh411)*, and *daf-10(m79); daf-12(rh61rh411)* animals on PA14. A–D. Survival curves of N2 (wild type), *daf-10(m79)*, *daf-12(rh61rh411)*, and *daf-10(m79); daf-12(rh61rh411)* animals on big lawns of *P. aeruginosa* PA14 are shown. In all cases, the survival curve of *daf-10(m79)* mutants crosses that of *daf-10(m79); daf-12(rh61rh411)* suggesting that hazard ratios are not constant. Thus, the log-rank test, which assumes constant hazard ratios, is not appropriate and we used the Wilcoxon test instead to determine significance. *p* values of the data presented in these panels are included in .
(EPS)

Figure S4 Alignment of the sequences of *mct-1* and *mct-2* (Wormbase WS230). The reference sequences for the genomic locus of *mct-1* and *mct-2* were aligned from 3 kb upstream of the translation start codons to 2 kb downstream of the translation stop codons. Promoter and untranslated regions are in black (exons of other genes are underlined), exons are in light blue and introns in dark blue. The red boxes highlight the limited differences in the sequence or in the splicing of the two genes. The positions of the primers used for qPCR and for cloning transgenic constructs are also marked.
(PDF)

Figure S5 Some canonical DAF-16/FOXO target genes are not differentially expressed in *daf-10(m79)* mutants. A. qRT-PCR analysis was used to examine the expression of two “canonical” targets of DAF-16/FOXO, *sod-3* and *mtl-1*, in *daf-10(m79)* animals. Neither of them was up-regulated in *daf-10(m79)* mutant animals.
(EPS)

Table S1 Lifespan analysis. Lifespan data within the solid bold lines include sets that were collected in parallel. In particular, within each panel of this table, lifespan data sets shaded in the same color were done in parallel. A. Effects of mutations in *daf-12* on the lifespan of *daf-10* and *daf-16; daf-10* mutants. *p* values and % changes for *daf-10(m79)* and *daf-12(rh61rh411)* single mutants were calculated against wild type, for *daf-10(m79); daf-12(rh61rh411)* mutants against *daf-12(rh61rh411)*, and for *daf-16(mu86); daf-10(m79); daf-12(rh61rh411)* mutants against *daf-16(mu86); daf-12(rh61rh411)* animals, respectively. Increase or decrease in lifespan are indicated as ‘+’ or ‘−’, respectively. *p* values in parentheses are marked and explained below. *^daf-10^*: *p* value against *daf-10(m79). ^$^*: These lifespan analyses were performed at 25°C. B. Effects of RNAi targeting genes up-regulated in *daf-10* mutants on the lifespan of various long-lived mutants including *daf-10* mutants. *p* values and % changes for the lifespan analysis of specific strains treated with *mct-1/2* RNAi were calculated against the same strains treated with control RNAi. *p* values and % changes in lifespan for single mutants or double mutants containing *rrf-3(pk1426)* mutation treated with control RNAi were calculated against wild type or *rrf-3(pk1426)* single mutant on control RNAi, respectively. Increase or decrease in lifespan were indicated as ‘+’ or ‘−’, respectively. *p* values in parentheses are marked and explained below. *^mct-1/2(RNAi)^*: *p* value against *mct-1/2(RNAi). ^rrf-3; mct-1/2(RNAi)^*: *p* value against *rrf-3(pk1426); mct-1/2(RNAi)*.
(DOCX)

Table S2 Statistical analysis of *P. aeruginosa* (PA14) resistance assay. Survival data within the solid bold lines include sets that were collected in parallel. In particular, within each panel of this table, data sets shaded in the same color were done in parallel. *p* values for single mutants were calculated against wild type and for double mutants against the corresponding single mutants (immediately above in the table) using the log-rank test (Mantel-Cox method) and the Wilcoxon test. *p* values in parentheses are marked and explained below. *^daf-l0^*: *p* value against *daf-10(m79)* mutant. *^daf-16(RNAi); rrf-3^*: *p* value against *daf-16(RNAi); rrf-3(pk1426). ^rrf-3; mct-1/2(RNAi)^*: *p* value against *rrf-3(pk1426); mct-1/2(RNAi)*. ^small^: *p* value against wild type on small lawn of PA14. ^#^: These pathogen resistance assays were carried out at 20°C.
(DOCX)

Table S3 Sequences of primers used for qRT–PCR analysis.
(DOCX)
