# Supplementary material for: Genes That Act Downstream of Sensory Neurons to Influence Longevity, Dauer Formation, and Pathogen Responses in Caenorhabditis elegans
Source: PLoS Genet. 2012 Dec 20;8(12):e1003133. doi: 10.1371/journal.pgen.1003133 (PMC3527274; doi:10.1371/journal.pgen.1003133)
Supplement: Figure S4 — Alignment of the sequences of mct-1 and mct-2 (Wormbase WS230). The reference sequences for the genomic locus of mct-1 and mct-2 were aligned from 3 kb upstream of the translation start codons to 2 kb downstream of the translation stop codons. Promoter and untranslated regions are in black (exons of other genes are underlined), exons are in light blue and introns in dark blue. The red boxes highlight the limited differences in the sequence or in the splicing of the two genes. The positions of the primers used for qPCR and for cloning transgenic constructs are also marked. (PDF) [file pgen.1003133.s004.pdf]

## Figure S4

Alignment of *mct-1* (Seq_1) and *mct-2* (Seq_2) from 3kb before the start codon to 2 kb after the stop codon

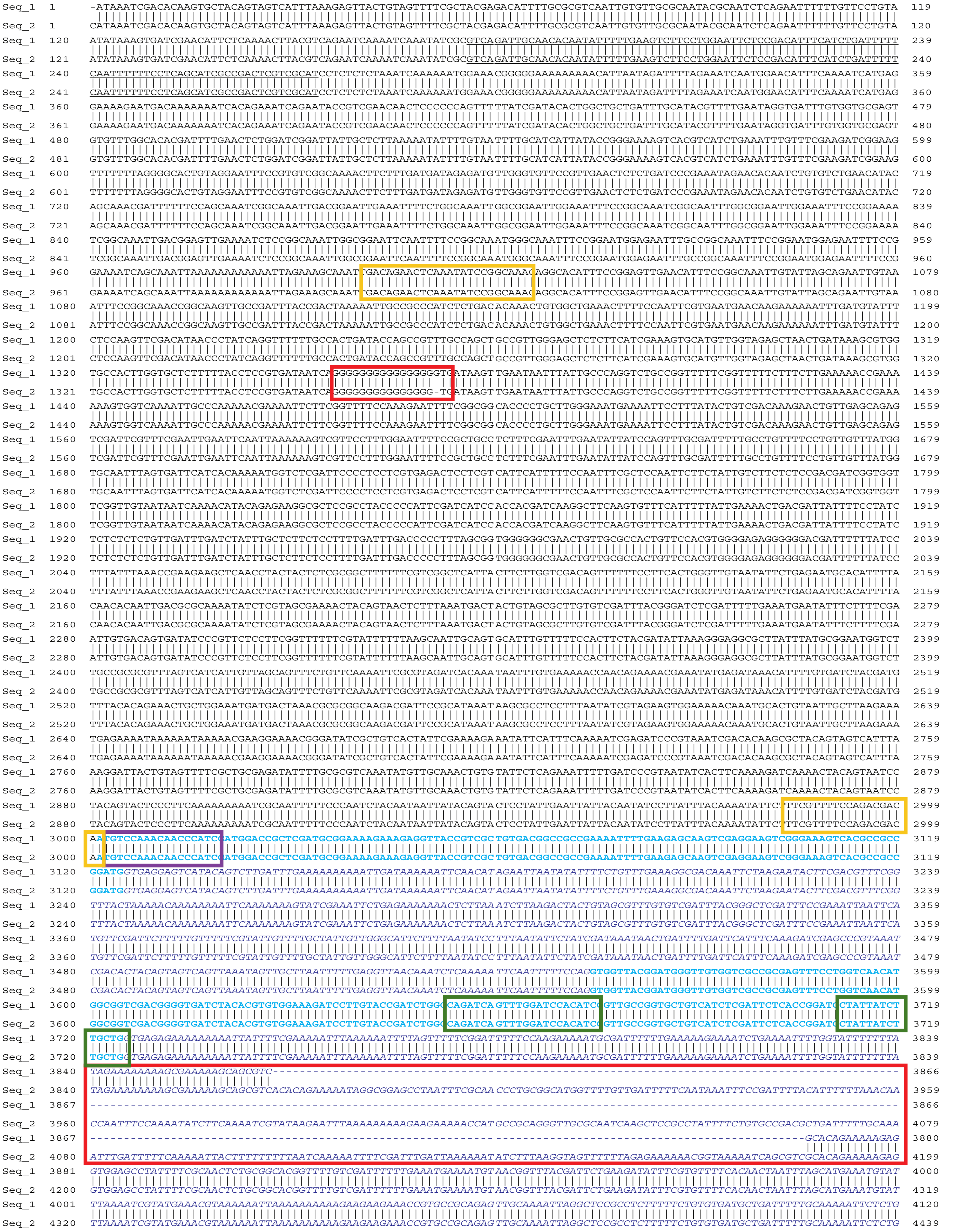

## Figure S4

Alignment of *mct-1* (Seq_1) and *mct-2* (Seq_2) from 3kb before the start codon to 2 kb after the stop codon

```
Seq_1  4121  AAATGAAAATTTTTGCATTTTTTGTTGAAAATATCAACAAAAAAAAACGATAAAATTTTTGATTTTCGAAAAAATAATTTTTTTTTAATTCCAACAAAAAAACCCCAATTTCAGGACCAC  4240
             ||||||||||||||||||||||||||||||||||||||||||||||||||||||||||||||||||||||||||||||||||||||||||||||||||||||||||||||||||||||||
Seq_2  4440  AAATGAAAATTTTTGCATTTTTTGTTGAAAATATCAACAAAAAAAAACGATAAAATTTTTGATTTTCGAAAAAATAATTTTTTTTTAATTCCAACAAAAAAACCCCAATTTCAGGACCAC  4559
Seq_1  4241  TGCCCTCGTCGTTCGTCAATGTGTTCGGAATCAGATCGGTAGCGATCGCCGGATCGATTCTCGCCACCACCGCCTTCCTCCTGTCCCGTTTCGCCAGCACAATCTGGGAACTTTATCTCC  4360
             ||||||||||||||||||||||||||||||||||||||||||||||||||||||||||||||||||||||||||||||||||||||||||||||||||||||||||||||||||||||||
Seq_2  4560  TGCCCTCGTCGTTCGTCAATGTGTTCGGAATCAGATCGGTAGCGATCGCCGGATCGATTCTCGCCACCACCGCCTTCCTCCTGTCCCGTTTCGCCAGCACAATCTGGGAACTTTATCTCC  4679
Seq_1  4361  TGTTCGGAGTTCTCGGTGGAATCGGTTTCGGATGTATGTATCTCCCGTCGATTGTCATTCTCTCGACATATTTCGCGAAAAAACGCAGCGTCGCCACCGGAATTGCCGTCTGCGGCTCGG  4480
             ||||||||||||||||||||||||||||||||||||||||||||||||||||||||||||||||||||||||||||||||||||||||||||||||||||||||||||||||||||||||
Seq_2  4680  TGTTCGGAGTTCTCGGTGGAATCGGTTTCGGATGTATGTATCTCCCGTCGATTGTCATTCTCTCGACATATTTCGCGAAAAAACGCAGCGTCGCCACCGGAATTGCCGTCTGCGGCTCGG  4799
Seq_1  4481  GAATCGGTACGATGGTCTTCTCGACGATCAACGGGCCCGTCTTTGATTATTTCGGTAAAGATGTCGGCTCATTTATGGTTTATCTGGCCGCAATTGCCATTTCCGGATCACTTTTCTCGC  4600
             ||||||||||||||||||||||||||||||||||||||||||||||||||||||||||||||||||||||||||||||||||||||||||||||||||||||||||||||||||||||||
Seq_2  4800  GAATCGGTACGATGGTCTTCTCGACGATCAACGGGCCCGTCTTTGATTATTTCGGTAAAGATGTCGGCTCATTTATGGTTTATCTGGCCGCAATTGCCATTTCCGGATCACTTTTCTCGC  4919
Seq_1  4601  TTCTTTTCGCTCCACTTAAAGCCACTGAACATCAGGTCAAAAAAGTCGCGAAAATGGTCAGAAATTACGAAGGAAAGCCGGAAGAGCCGACACAGAGACTGCTCGAAGATGTTCGTAACG  4720
             ||||||||||||||||||||||||||||||||||||||||||||||||||||||||||||||||||||||||||||||||||||||||||||||||||||||||||||||||||||||||
Seq_2  4920  TTCTTTTCGCTCCACTTAAAGCCACTGAACATCAGGTCAAAAAAGTCGCGAAAATGGTCAGAAATTACGAAGGAAAGCCGGAAGAGCCGACACAGAGACTGCTCGAAGATGTTCGTAACG  5039
Seq_1  4721  ATTTGGAAGAGCTCAATCGCCCCGGACACAATGCTGACACTTTTTACGCCGGAAATGCTCCAGTTAGCAGAAGCCGTTCCAATACTTGTTCGTTTTTGGGAATTTTTCTTTTTAAAAAAA  4840
             ||||||||||||||||||||||||||||||||||||||||||||||||||||||||||||||||||||||||||||||||||||||||||||||||||||||||||||||||||||||||
Seq_2  5040  ATTTGGAAGAGCTCAATCGCCCCGGACACAATGCTGACACTTTTTACGCCGGAAATGCTCCAGTTAGCAGAAGCCGTTCCAATACTTGTTCGTTTTTGGGAATTTTTCTTTTTAAAAAAA  5159
Seq_1  4841  TCCAAAATTGTCTCAAATTCAGAGAAACCATAAAAAAACAGGAAAAAAACGTGATTTTTCAATTTTTAGCTAAATTCCCTCCAAAAGGAGAACTGCAACTTATTACGGGATCGCAAAATTTT  4960
             ||||||||||||||||||||||||||||||||||||||||||||||||||||||||||||||||||||||||||||||||||||||||||||||||||||||||||||||||||||||||
Seq_2  5160  TCCAAAATTGTCTCAAATTCAGAGAAACCATAAAAAAACAGGAAAAAAACGTGATTTTTCAATTTTTAGCTAAATTCCCTCCAAAAGGAGAACTGCAACTTATTACGGGATCGCAAAATTTT  5279
Seq_1  4961  GAGAATGCGTATTACACAACATATTTGACGCGCAAAATATCTCGTAGCGAAAACTACAGTAATCCTGTAAATGACGACTGTATTGCTGCTGATGTCGATTTACGGGGCTCGATTTTCGAA  5080
             ||||||||||||||||||||||||||||||||||||||||||||||||||||||||||||||||||||||||||||||||||||||||||||||||||||||||||||||||||||||||
Seq_2  5280  GAGAATGCGTATTACACAACATATTTGACGCGCAAAATATCTCGTAGCGAAAACTACAGTAATCCTGTAAATGACGACTGTATTGCTGCTGATGTCGATTTACGGGGCTCGATTTTCGAA  5399
Seq_1  5081  ATAATTTCTTTTTCGAATAGTGACAGCGTAATTTTAATGCAATATCGCTGTCACAATTCGCAAAAAAAAAAACCATTTCAAAATTCGATATCCCGTAAATCGACACAAGCGCTACAGTAAC  5200
             ||||||||||||||||||||||||||||||||||||||||||||||||||||||||||||||||||||||||||||||||||||||||||||||||||||||||||||||||||||||||
Seq_2  5400  ATAATTTCTTTTTCGAATAGTGACAGCGTAATTTTAATGCAATATCGCTGTCACAATTCGCAAAAAAAAAAACCATTTCAAAATTCGATATCCCGTAAATCGACACAAGCGCTACAGTAAC  5519
Seq_1  5201  CATTCAAAGGGTTACTGTAGTATTCGCTACGGGATATTTTGCGCGTCAAATATGTTGCAAAATGTGCATTCTCAGAATTTTCAGCTTCCGTAATAGGATACAGTTTTCATTTTGGAGGGA  5320
             ||||||||||||||||||||||||||||||||||||||||||||||||||||||||||||||||||||||||||||||||||||||||||||||||||||||||||||||||||||||||
Seq_2  5520  CATTCAAAGGGTTACTGTAGTATTCGCTACGGGATATTTTGCGCGTCAAATATGTTGCAAAATGTGCATTCTCAGAATTTTCAGCTTCCGTAATAGGATACAGTTTTCATTTTGGAGGGA  5639
Seq_1  5321  ATTTTGGCCAAAATTTAAAAAAATCGAGTATTTTTTGAATTTGAGGCAATTTTGATTTTTTTTTTTTTTGAAAGCTTGAAGCTAAATCATGAGGACCCACGAAAAGGGGTCTGGCACTGTGC  5440
             ||||||||||||||||||||||||||||||||||||||||||||||||||||||||||||||||||||||||||||||||||||||||||||||||||||||||||||||||||||||||
Seq_2  5640  ATTTTGGCCAAAATTTAAAAAAATCGAGTATTTTTTGAATTTGAGGCAATTTTGATTTTTTTTTTTTTTGAAAGCTTGAAGCTAAATCATGAGGACCCACGAAAAGGGGTCTGGCACTGTGC  5759
Seq_1  5441  CAACGCACAAAACATACTTTTTCTCAAAGAAAACGATACAACGATGCTCCGAAATTACGCGTCGCGCGTTGTTAAGCGTATCTTCTAGAAGAATTTCGAAAAAAGTCGTGCGTTAAATGA  5560
             ||||||||||||||||||||||||||||||||||||||||||||||||||||||||||||||||||||||||||||||||||||||||||||||||||||||||||||||||||||||||
Seq_2  5760  CAACGCACAAAACATACTTTTTCTCAAAGAAAACGATACAACGATGCTCCGAAATTACGCGTCGCGCGTTGTTAAGCGTATCTTCTAGAAGAATTTCGAAAAAAGTCGTGCGTTAAATGA  5879
Seq_1  5561  ATAAAAAAGCGTTTTGTGCGTTGGCACAGTGACAATTTCCCCTTTTCGTGGGCCCTCGTTTTCTTCGAGAAAAAATCGCGTTTTATGCGTTGGCACTGTGCTAGATCACCTTTTTGCGAAT  5680
             ||||||||||||||||||||||||||||||||||||||||||||||||||||||||||||||||||||||||||||||||||||||||||||||||||||||||||||||||||||||||
Seq_2  5880  ATAAAAAAGCGTTTTGTGCGTTGGCACAGTGACAATTTCCCCTTTTCGTGGGCCCTCGTTTTCTTCGAGAAAAAATCGCGTTTTATGCGTTGGCACTGTGCTAGATCACCTTTTTGCGAAT  5999
Seq_1  5681  TTCCTTTCCGTGGGGCCTCTAAATAATCTCCAAAAAATCTCAATTTTTCATTTTCAGTGGACCGGAAAGCCGCCGAATCTGCTGAAGCTCATGTCGTCCACGCCACCGAACACCACACCG  5800
             ||||||||||||||||||||||||||||||||||||||||||||||||||||||||||||||||||||||||||||||||||||||||||||||||||||||||||||||||||||||||
Seq_2  6000  TTCCTTTCCGTGGGGCCTCTAAATAATCTCCAAAAAATCTCAATTTTTCATTTTCAGTGGACCGGAAAGCCGCCGAATCTGCTGAAGCTCATGTCGTCCACGCCACCGAACACCACACCG  6119
Seq_1  5801  TCCATCATGTCGTCAAGAAGAGCAAATTCACCAAATTCAAGGAATCACTGTGCTCTGTGCTCGACAAGGATCTTCTCTTCTCTCCATCATTCATGACTCTTGCCGTCTCAGGAACTTTCA  5920
             ||||||||||||||||||||||||||||||||||||||||||||||||||||||||||||||||||||||||||||||||||||||||||||||||||||||||||||||||||||||||
Seq_2  6120  TCCATCATGTCGTCAAGAAGAGCAAATTCACCAAATTCAAGGAATCACTGTGCTCTGTGCTCGACAAGGATCTTCTCTTCTCTCCATCATTCATGACTCTTGCCGTCTCAGGAACTTTCA  6239
Seq_1  5921  CCGTCCTCTCGTTCCTTGTTCCATTCGTCTACTTGGCTCTTGCCATGAAGCAGAAGAATCCGGATTTTACTGATGCTGAATTGTCACTTCCAGTCACTCTAATCGGTGCTTTCAACATTA  6040
             ||||||||||||||||||||||||||||||||||||||||||||||||||||||||||||||||||||||||||||||||||||||||||||||||||||||||||||||||||||||||
Seq_2  6240  CCGTCCTCTCGTTCCTTGTTCCATTCGTCTACTTGGCTCTTGCCATGAAGCAGAAGAATCCGGATTTTACTGATGCTGAATTGTCACTTCCAGTCACTCTAATCGGTGCTTTCAACATTA  6359
Seq_1  6041  TGTTCCGTATCGGATGTGGAATGGTCGCTGATCATCCGAAAATGTCGGCTCTTCAAGTTTCCAACGTCGCCACCATCATCGCCGGAACCTCGATGCTCTTTGTGCCATTTTGCACTGAGC  6160
             ||||||||||||||||||||||||||||||||||||||||||||||||||||||||||||||||||||||||||||||||||||||||||||||||||||||||||||||||||||||||
Seq_2  6360  TGTTCCGTATCGGATGTGGAATGGTCGCTGATCATCCGAAAATGTCGGCTCTTCAAGTTTCCAACGTCGCCACCATCATCGCCGGAACCTCGATGCTCTTTGTGCCATTTTGCACTGAGC  6479
Seq_1  6161  TCTGGCATTACGTCGTTTTCTGCATCCCGTTCAGTGCCGGAGTTGGTGAGTTTTGGAGAAAAATCCCGTTGCTTTTAACGATTTTTACGTCGAAATTGAAAAAAAAGATGTTTAAAATAT  6280
             ||||||||||||||||||||||||||||||||||||||||||||||||||||||||||||||||||||||||||||||||||||||||||||||||||||||||||||||||||||||||
Seq_2  6480  TCTGGCATTACGTCGTTTTCTGCATCCCGTTCAGTGCCGGAGTTGGTGAGTTTTGGAGAAAAATCCCGTTGCTTTTAACGATTTTTACGTCGAAATTGAAAAAAAAGATGTTTAAAATAT  6599
Seq_1  6281  CAATTTCGGACTTAAAATATCAATAACCACAAAAAAATTTGCGAAATTAAGGTGGAAACGCGCTCCACGGACAATCGGTAATACTCCGCCCACAAAATTTGGGTCTCGTTAGGTATTTGAC  6400
             ||||||||||||||||||||||||||||||||||||||||||||||||||||||||||||||||||||||||||||||||||||||||||||||||||||||||||||||||||||||||
Seq_2  6600  CAATTTCGGACTTAAAATATCAATAACCACAAAAAAATTTGCGAAATTAAGGTGGAAACGCGCTCCACGGACAATCGGTAATACTCCGCCCACAAAATTTGGGTCTCGTTAGGTATTTGAC  6719
Seq_1  6401  AGAAAAGTCGTATTTTCGAGCATTTTTCATTTTTATTGTTAAATTTTTCGTTGTTTTTATTGATTTTTATGTCGAAATTGATAAAATAATATTTATAATTTTATTTCATTAATTTCAGCT  6520
             ||||||||||||||||||||||||||||||||||||||||||||||||||||||||||||||||||||||||||||||||||||||||||||||||||||||||||||||||||||||||
Seq_2  6720  AGAAAAGTCGTATTTTCGAGCATTTTTCATTTTTATTGTTAAATTTTTCGTTGTTTTTATTGATTTTTATGTCGAAATTGATAAAATAATATTTATAATTTTATTTCATTAATTTCAGCT  6839
Seq_1  6521  TAAAATCGATAAGAAAAACAATGGAAAAATGACGAAAAAAGCGAAAATTGGTTGATTCTGGCCTCAATTTGCCGGTTTTAATTTTTTTTTTTGCCGATTTCCCCTGTTTTCTATCATTTT  6640
             ||||||||||||||||||||||||||||||||||||||||||||||||||||||||||||||||||||||||||||||||||||||||||||||||||||||||||||||||||||||||
Seq_2  6840  TAAAATCGATAAGAAAAACAATGGAAAAATGACGAAAAAAGCGAAAATTGGTTGATTCTGGCCTCAATTTGCCGGTTTTAATTTTTTTTTTTGCCGATTTCCCCTGTTTTCTATCATTTT  6959
Seq_1  6641  TCGTTGTTTTAATAATTTTCACGTCGAAATTGAATTAAAAAATTTAATTTCGGCTTAAAAATCACAAAAACTACCTAAAAAAACGGCGAAAAATGCTTGAAAATTGGCAAACTATTTTAAA  6760
             ||||||||||||||||||||||||||||||||||||||||||||||||||||||||||||||||||||||||||||||||||||||||||||||||||||||||||||||||||||||||
Seq_2  6960  TCGTTGTTTTAATAATTTTCACGTCGAAATTGAATTAAAAAATTTAATTTCGGCTTAAAAATCACAAAAACTACCTAAAAAAACGGCGAAAAATGCTTGAAAATTGGCAAACTATTTTAAA  7079
Seq_1  6761  AATTTCAAATTTGCGTTTTTATCGCCAAATACCCGATGAGACCCAAATTTTTGGAGGTGGAGTGTTGTCGATTGTCCGTGGAGCGCGTTTTTTCACGAAAAAAAAAACCAAAAATTAAAA  6880
             ||||||||||||||||||||||||||||||||||||||||||||||||||||||||||||||||||||||||||||||||||||||||||||||||||||||||||||||||||||||||
Seq_2  7080  AATTTCAAATTTGCGTTTTTATCGCCAAATACCCGATGAGACCCAAATTTTTGGAGGTGGAGTGTTGTCGATTGTCCGTGGAGCGCGTTTTTTCACGAAAAAAAAAACCAAAAATTAAAA  7199
Seq_1  6881  AAAAACTTAAATATTTAAAAAAATCAGTTAAAAAAATTTCGAGCGTGTTTTTGAAAAAAAAAAAGTAATCAGTTTTTCTTTTTAAAAATCCAAAAAAAAGCGAATGTCCCCATCAAAGGA  7000
             ||||||||||||||||||||||||||||||||||||||||||||||||||||||||||||||||||||||||||||||||||||||||||||||||||||||||||||||||||||||||
Seq_2  7200  AAAAACTTAAATATTTAAAAAAATCAGTTAAAAAAATTTCGAGCGTGTTTTTGAAAAAAAAAAAGTAATCAGTTTTTCTTTTTAAAAATCCAAAAAAAAGCGAATGTCCCCATCAAAGGA  7319
Seq_1  7001  GCCACCGAAAATTTTTTTAAATACTTTTTTGAACGAATTTGGCCCCAATTTTTTTCTGAAAAAAAACGATAAATCTTCGGTTTTGTCGAAAAAAAAAAATCGAAAAACCGACACCCTTGGT  7120
             ||||||||||||||||||||||||||||||||||||||||||||||||||||||||||||||||||||||||||||||||||||||||||||||||||||||||||||||||||||||||
Seq_2  7320  GCCACCGAAAATTTTTTTAAATACTTTTTTGAACGAATTTGGCCCCAATTTTTTTCTGAAAAAAAACGATAAATCTTCGGTTTTGTCGAAAAAAAAAAATCGAAAAACCGACACCCTTGGT  7439
Seq_1  7121  CCCCATATTAAATTAATAAATCTTGAACTATTTCTCCATTTCAGCTTGCTTCGCCGCTCTTCGTTCGGTCATCTGCGTCGAGCTGATCGGAGTCGAAAAGCTGTCGAACGCGTTCGGCAT  7240
             ||||||||||||||||||||||||||||||||||||||||||||||||||||||||||||||||||||||||||||||||||||||||||||||||||||||||||||||||||||||||
Seq_2  7440  CCCCATATTAAATTAATAAATCTTGAACTATTTCTCCATTTCAGCTTGCTTCGCCGCTCTTCGTTCGGTCATCTGCGTCGAGCTGATCGGAGTCGAAAAGCTGTCGAACGCGTTCGGCAT  7559
Seq_1  7241  CCTGATGGTTTTCATGGGAATCGGCGCTGTCGTCGGCGGGCCGATTGCCGCTCAAATCAAGGATATCACTGGCAACTACGACATTAGCTTCTACGTGATGGGCATCATTTTCGCCTTCTC  7360
             ||||||||||||||||||||||||||||||||||||||||||||||||||||||||||||||||||||||||||||||||||||||||||||||||||||||||||||||||||||||||
Seq_2  7560  CCTGATGGTTTTCATGGGAATCGGCGCTGTCGTCGGCGGGCCGATTGCCGCTCAAATCAAGGATATCACTGGCAACTACGACATTAGCTTCTACGTGATGGGCATCATTTTCGCCTTCTC  7679
Seq_1  7361  CGGCGTCATGACTATCCGTTTGCCAGAGCTGAAGGCGTGGGAAGAGTCGAAAAAACTCGCACGCGCCGGAACCGAGATGCGTGTGATCTCTGAAGCATCCTAATTTTTTGTTTTAAAAAT  7480
             ||||||||||||||||||||||||||||||||||||||||||||||||||||||||||||||||||||||||||||||||||||||||||||||||||||||||||||||||||||||||
Seq_2  7680  CGGCGTCATGACTATCCGTTTGCCAGAGCTGAAGGCGTGGGAAGAGTCGAAAAAACTCGCACGCGCCGGAACCGAGATGCGTGTGATCTCTGAAGCATCCTAATTTTTTGTTTTAAAAAT  7799
Seq_1  7481  ATCTCAATTTTTTTTTTAAATTTTTGTTTGTTTGAATATCCTATTCCTTTAATTCTAATTCCGATTTTTATCTGTTTCCCCATTTTCCCCCCAAAAAGTTCCGAAGAGTTCAACTCTCTC  7600
             ||||||||||||||||||||||||||||||||||||||||||||||||||||||||||||||||||||||||||||||||||||||||||||||||||||||||||||||||||||||||
Seq_2  7800  ATCTCAATTTTTTTTTTAAATTTTTGTTTGTTTGAATATCCTATTCCTTTAATTCTAATTCCGATTTTTATCTGTTTCCCCATTTTCCCCCCAAAAAGTTCCGAAGAGTTCAACTCTCTC  7919
Seq_1  7601  ATCTCCATCCCTCAAGCATTTTTTTTTTCAAATAGGTGCCTTTTTCACGTGGTGACAGGCTGTCCCATTACAGTTTGATCTACAAAAAATGCGGGAATTTTTTGATCAAAAAGATTTGACG  7720
             ||||||||||||||||||||||||||||||||||||||||||||||||||||||||||||||||||||||||||||||||||||||||||||||||||||||||||||||||||||||||
Seq_2  7920  ATCTCCATCCCTCAAGCATTTTTTTTTTCAAATAGGTGCCTTTTTCACGTGGTGACAGGCTGTCCCATTACAGTTTGATCTACAAAAAATGCGGGAATTTTTTGATCAAAAAGATTTGACG  8039
Seq_1  7721  TCATCACGTTCTTAACCAATCAGTTGGGTAGTCTGCGTCTCTCTTTTCCCGCATTTTTTGTAGATCAAGTCTGCCACCACGTTTTTTCCGCTTTTTTGCCACCGGTTGTCATCACTTCCA  7840
             ||||||||||||||||||||||||||||||||||||||||||||||||||||||||||||||||||||||||||||||||||||||||||||||||||||||||||||||||||||||||
Seq_2  8040  TCATCACGTTCTTAACCAATCAGTTGGGTAGTCTGCGTCTCTCTTTTCCCGCATTTTTTGTAGATCAAGTCTGCCACCACGTTTTTTCCGCTTTTTTGCCACCGGTTGTCATCACTTCCA  8159
Seq_1  7841  CCCCATCAAATGAATTCACTCAGTTCCCGCACACTGACTTTTTTAATCTCTTTTTGAATAATTGTCCAAGATTGTTAATTAATTTCATTAATAACCACTGATAATTGATAATTGAGCTAG  7960
             ||||||||||||||||||||||||||||||||||||||||||||||||||||||||||||||||||||||||||||||||||||||||||||||||||||||||||||||||||||||||
Seq_2  8160  CCCCATCAAATGAATTCACTCAGTTCCCGCACACTGACTTTTTTAATCTCTTTTTGAATAATTGTCCAAGATTGTTAATTAATTTCATTAATAACCACTGATAATTGATAATTGAGCTAG  8279
Seq_1  7961  TAATAAAGTCACAGTTTTTTTCTCAAAAATTGTAACTTTTTGCCATTTTTTTCTCTCTCTTTCCCCGACCATTCGATCATCATCATCACCAACTATCACTGTTCACTTCCGCTGATGCAC  8080
             ||||||||||||||||||||||||||||||||||||||||||||||||||||||||||||||||||||||||||||||||||||||||||||||||||||||||||||||||||||||||
Seq_2  8280  TAATAAAGTCACAGTTTTTTTCTCAAAAATTGTAACTTTTTGCCATTTTTTTCTCTCTCTTTCCCCGACCATTCGATCATCATCATCACCAACTATCACTGTTCACTTCCGCTGATGCAC  8399
Seq_1  8081  CATGCCTCCTGGAATATTCAAATGAGGGTACACAGAAGTCACGAATTCACGGAGGACTACACGAGTCTAGTACGACTTATAAAAACTTTGAAATGTCATGGTTTTTCCAGATTTTTCCAG  8200
             ||||||||||||||||||||||||||||||||||||||||||||||||||||||||||||||||||||||||||||||||||||||||||||||||||||||||||||||||||||||||
Seq_2  8400  CATGCCTCCTGGAATATTCAAATGAGGGTACACAGAAGTCACGAATTCACGGAGGACTACACGAGTCTAGTACGACTTATAAAAACTTTGAAATGTCATGGTTTTTCCAGATTTTTCCAG  8519
Seq_1  8201  TTCAAATGGCATTTTTTTGGTGAAATTTCTGCTTATTATGTGGACGCAGAAAAATGTGTTTTTTTGCAAGCGCGCTCCACGGCTAATCACCAAACGCTGCGTTCTCCGGGTCTCGTTAGG  8320
             ||||||||||||||||||||||||||||||||||||||||||||||||||||||||||||||||||||||||||||||||||||||||||||||||||||||||||||||||||||||||
Seq_2  8520  TTCAAATGGCATTTTTTTGGTGAAATTTCTGCTTATTATGTGGACGCAGAAAAATGTGTTTTTTTGCAAGCGCGCTCCACGGCTAATCACCAAACGCTGCGTTCTCCGGGTCTCGTTAGG  8639
```

**Figure S4**

Alignment of *mct-1* (Seq_1) and *mct-2* (Seq_2) from 3kb before the start codon to 2 kb after the stop codon

```
Seq_1  8321  AATTTGGCGGCGAATTTGTTGAATTTTGCAATTTTTGTTAGGTTTTCCGTTTTTTTAGTGATTTTTATGTCGAAATTGAGAAAAAAAAATGTTTAAAAATTCGATTTCGGAATAAAAATCA  8440
             ||||||||||||||||||||||||||||||||||||||||||||||||||||||||||||||||||||||||||||||||||||||||||||||||||||||||||||||||||||||||
Seq_2  8640  AATTTGGCGGCGAATTTGTTGAATTTTGCAATTTTTGTTAGGTTTTCCGTTTTTTTAGTGATTTTTATGTCGAAATTGAGAAAAAAAAATGTTTAAAAATTCGATTTCGGAATAAAAATCA  8759

Seq_1  8441  CAAAAAGTTGTGAAAACGCGCTCCACGGACAATCGATAACACTCCGCCTCTAAAAATTGGGTCTCGTTAGGTTTTTGGCACAGAAAAACCGTTTTTTCGACGATTTCCAAGCATTTTTCA  8560
             ||||||||||||||||||||||||||||||||||||||||||||||||||||||||||||||||||||||||||||||||||||||||||||||||||||||||||||||||||||||||
Seq_2  8760  CAAAAAGTTGTGAAAACGCGCTCCACGGACAATCGATAACACTCCGCCTCTAAAAATTGGGTCTCGTTAGGTTTTTGGCACAGAAAAACCGTTTTTTCGACGATTTCCAAGCATTTTTCA  8879

Seq_1  8561  CTTTTTTTGTTAAGTTTTCCGTTGTTTTTAGTGATTTTTACGTCGAAATGTACAAAAAAAGCTCAAAACTTTAATTTCAACGTGAAAATCATTAAACACTCGACAAAAACCCCGAAAAAT  8680
             ||||||||||||||||||||||||||||||||||||||||||||||||||||||||||||||||||||||||||||||||||||||||||||||||||||||||||||||||||||||||
Seq_2  8880  CTTTTTTTGTTAAGTTTTCCGTTGTTTTTAGTGATTTTTACGTCGAAATGTACAAAAAAAGCTCAAAACTTTAATTTCAACGTGAAAATCATTAAACACTCGACAAAAACCCCGAAAAAT  8999

Seq_1  8681  GACAGAAAACTTTTGAATTTACGGTTTTGCCGCCAAAGGCCAGTGGAGCGCACTTGCACTTCTGTTTTAAAATCGGCGTGTTTTCCCACACTGTATTTGTTTTTCCACTTCTACACTTAA  8800
             ||||||||||||||||||||||||||||||||||||||||||||||||||||||||||||||||||||||||||||||||||||||||||||||||||||||||||||||||||||||||
Seq_2  9000  GACAGAAAACTTTTGAATTTACGGTTTTGCCGCCAAAGGCCAGTGGAGCGCACTTGCACTTCTGTTTTAAAATCGGCGTGTTTTCCCACACTGTATTTGTTTTTCCACTTCTACACTTAA  9119

Seq_1  8801  AGGATGACGTTTTTATACGAAATGGTCTCGCCACGTGCCCCAAAAATCAGGCGCGTGGTGAGACCATTTCGTATAAACTCCTTTAAAGGCGGAGAAGTGGAAAAATTGTCTCATTTCGGT  8920
             ||||||||||||||||||||||||||||||||||||||||||||||||||||||||||||||||||||||||||||||||||||||||||||||||||||||||||||||||||||||||
Seq_2  9120  AGGATGACGTTTTTATACGAAATGGTCTCGCCACGTGCCCCAAAAATCAGGCGCGTGGTGAGACCATTTCGTATAAACTCCTTTAAAGGCGGAGAAGTGGAAAAATTGTCTCATTTCGGT  9239

Seq_1  8921  TAGATCTACGTAGATCTACAAAAAATGCGGCAGAGTTCTCAACTGATTTCGCATGGTTAAGAACGTGCTGACGTCACATTTTTTTGGGCGAAAAATTCCCGCTTTTTTGTAGATCAAACC  9040
             ||||||||||||||||||||||||||||||||||||||||||||||||||||||||||||||||||||||||||||||||||||||||||||||||||||||||||||||||||||||||
Seq_2  9240  TAGATCTACGTAGATCTACAAAAAATGCGGCAGAGTTCTCAACTGATTTCGCATGGTTAAGAACGTGCTGACGTCACATTTTTTTGGGCGAAAAATTCCCGCTTTTTTGTAGATCAAACC  9359

Seq_1  9041  GTAATGGGACAGTCTGGCACCTCGTGATAAGTTTGCGGTTAAGTAAATAAAAATTAAATTTTTAGGAAGCTTTCTCAGAAATGGGACAAAGCTCGGAAGTTCACCCGGAATTTGGATTCC  9160
             ||||||||||||||||||||||||||||||||||||||||||||||||||||||||||||||||||||||||||||||||||||||||||||||||||||||||||||||||||||||||
Seq_2  9360  GTAATGGGACAGTCTGGCACCTCGTGATAAGTTTGCGGTTAAGTAAATAAAAATTAAATTTTTAGGAAGCTTTCTCAGAAATGGGACAAAGCTCGGAAGTTCACCCGGAATTTGGATTCC  9479

Seq_1  9161  CACAAATTCGGAAAACGTCAAGTTTTGTATTCTGGAATCGGACGCGGCTTTATATTATGGTTTTGACGTGGGTTTTATTTTTTTTTTAATCGATAAAATATCTCCATATCTGGGGTGGAT  9280
             ||||||||||||||||||||||||||||||||||||||||||||||||||||||||||||||||||||||||||||||||||||||||||||||||||||||||||||||||||||||||
Seq_2  9480  CACAAATTCGGAAAACGTCAAGTTTTGTATTCTGGAATCGGACGCGGCTTTATATTATGGTTTTGACGTGGGTTTTATTTTTTTTTTAATCGATAAAATATCTCCATATCTGGGGTGGAT  9599

Seq_1  9281  TTACGGCGCGTTGCGTGTCGCGTCGCGGCTCGATTTTAGTTGTAAGACTATAGTTATTTGTCCGTGTGGAGTTCACGACACTTTTCCACGCGTTGTCCGACTGGCGATTGTCAATGGAGC  9400
             ||||||||||||||||||||||||||||||||||||||||||||||||||||||||||||||||||||||||||||||||||||||||||||||||||||||||||||||||||||||||
Seq_2  9600  TTACGGCGCGTTGCGTGTCGCGTCGCGGCTCGATTTTAGTTGTAAGACTATAGTTATTTGTCCGTGTGGAGTTCACGACACTTTTCCACGCGTTGTCCGACTGGCGATTGTCAATGGAGC  9719

Seq_1  9401  GCGAAAAACGTAACGAGGGAGGCCAGGAGCCCGTGATTAAATATTTCATTTCTTCAAATTTTG  9463
             |||||||||||||||||||||||||||||||||||||||||||||||||||||||||||||||
Seq_2  9720  GCGAAAAACGTAACGAGGGAGGCCAGGAGCCCGTGATTAAATATTTCATTTCTTCAAATTTTG  9782
```

**light blue** = exons

*dark blue* = introns

black (underlined) = exons of other genes

red boxes = differences between two sequences

purple boxes = promoter cloning primers

yellow boxes = cDNA cloning primers

green boxes = qPCR primers (reverse across exon 2-3 junction)
