# Supplementary material for: Genes That Act Downstream of Sensory Neurons to Influence Longevity, Dauer Formation, and Pathogen Responses in Caenorhabditis elegans
Source: PLoS Genet. 2012 Dec 20;8(12):e1003133. doi: 10.1371/journal.pgen.1003133 (PMC3527274; doi:10.1371/journal.pgen.1003133)
Supplement: Table S1 — Lifespan analysis. Lifespan data within the solid bold lines include sets that were collected in parallel. In particular, within each panel of this table, lifespan data sets shaded in the same color were done in parallel. A. Effects of mutations in daf-12 on the lifespan of daf-10 and daf-16; daf-10 mutants. p values and % changes for daf-10(m79) and daf-12(rh61rh411) single mutants were calculated against wild type, for daf-10(m79); daf-12(rh61rh411) mutants against daf-12(rh61rh411), and for daf-16(mu86); daf-10(m79); daf-12(rh61rh411) mutants against daf-16(mu86); daf-12(rh61rh411) animals, respectively. Increase or decrease in lifespan are indicated as ‘+’ or ‘−’, respectively. p values in parentheses are marked and explained below. daf-10: p value against daf-10(m79). $: These lifespan analyses were performed at 25°C. B. Effects of RNAi targeting genes up-regulated in daf-10 mutants on the lifespan of various long-lived mutants including daf-10 mutants. p values and % changes for the lifespan analysis of specific strains treated with mct-1/2 RNAi were calculated against the same strains treated with control RNAi. p values and % changes in lifespan for single mutants or double mutants containing rrf-3(pk1426) mutation treated with control RNAi were calculated against wild type or rrf-3(pk1426) single mutant on control RNAi, respectively. Increase or decrease in lifespan were indicated as ‘+’ or ‘−’, respectively. p values in parentheses are marked and explained below. mct-1/2(RNAi): p value against mct-1/2(RNAi). rrf-3; mct-1/2(RNAi): p value against rrf-3(pk1426); mct-1/2(RNAi). (DOCX) [file pgen.1003133.s006.docx]

**Table S1. Lifespan analysis**

**A.** Effects of mutations in *daf-12* on the lifespan of *daf-10* and *daf-16; daf-10* mutants.

| **Strain/treatment** | **Mean lifespan ±s.e.m. (days)** | **75th percentile** | **%**  **change** | **Number of animals that died/total** | ***P* value vs. control** | **Figure in text** |
| --- | --- | --- | --- | --- | --- | --- |
| Wild type | 23.0±0.9 | 28 |  | 32/237 |  | Fig. 2B |
|  | 17.9±0.6 | 22 |  | 58/115 |  |  |
| *^$^* | 9.9±0.2 | 12 |  | 171/180 |  | Fig. 2C |
| *^$^* | 9.9±0.2 | 12 |  | 163/180 |  |  |
| *daf-10(m79)* | 36.9±0.5 | 39 | +60% | 99/181 | <0.0001 | Fig. 2B |
|  | 30.3±0.6 | 34 | -3% | 106/176 | <0.0001 |  |
| *^$^* | 14.8±0.3 | 19 | +49.6% | 162/180 | <0.0001 | Fig. 2C |
| *^$^* | 13.0±0.3 | 14 | +31.7% | 165/180 | <0.0001 |  |
| *daf-12(rh61rh411)* | 23.2±1.3 | 28 | +1% | 35/181 | 0.9508 | Fig. 2B |
|  | 17.3±0.8 | 20 | +69.1% | 64/125 | 0.7716 |  |
| *^$^* | 10.2±0.2 | 12 | +2.8% | 174/180 | 0.3509 | Fig. 2C |
| *^$^* | 9.9±0.2 | 12 | +0.3% | 137/180 | 0.8813 |  |
| *daf-10(m79); daf-12(rh61rh411)* | 35.1±0.9 | 39 | +51.3% | 45/173 | <0.0001 (0.0847 *^daf-10^*) | Fig. 2B |
|  | 30.2±1.0 | 36 | +74% | 72/181 | <0.0001(0.274 *^daf-10^*) |  |
| *^$^* | 15.3±0.3 | 19 | +50.5% | 144/150 | <0.0001  (0.6099 *^daf-10^*) | Fig. 2C |
| *^$^* | 13.2±0.3 | 16 | +33.6% | 165/180 | <0.0001  (0.8614 *^daf-10^*) |  |
| *daf-16(mu86); daf-12(rh61rh411)* | 13.1±0.4 | 15 |  | 86/130 |  | Fig. 2D |
| *daf-16(mu86); daf-10(m79); daf-12(rh61rh411)* | 16.3±0.7 | 22 | +24.4% | 56/129 | <0.001 | Fig. 2D |

**B.** Effects of RNAi targeting genes up-regulated in *daf-10* mutants on the lifespan of various long-lived mutants including *daf-10* mutants.

| **Strain/treatment** | **Mean lifespan ±s.e.m. (days)** | **75th percentile** | **%**  **change** | **Number of animals that died/total** | ***P* value vs. control** | **Figure in text** |
| --- | --- | --- | --- | --- | --- | --- |
| *rrf-3(pk1426); daf-10(m79)* | 23.5±1.1 | 30 |  | 60/84 |  | Fig. 5A |
| *daf-16*(*RNAi*);  *rrf-3(pk1426); daf-10(m79)* | 18.7±0.6 | 21 | -20.4% | 47/72 | <0.001 | Fig. 5A |
| *rrf-3(pk1426); daf-10(m79);*  *mct-1/2(RNAi)* | 16.5±0.4 | 19 | -29.8% | 49/80 | <0.0001 | Fig. 5A |
| *rrf-3(pk1426); daf-10(m79); W02D7.8(RNAi)* | 25.7±1.1 | 33 | +9.4% | 39/85 | 0.367 | Fig. 5A |
| *rrf-3(pk1426); daf-10(m79);* *F14D7.7(RNAi)* | 26.0±1.1 | 30 | +10.6% | 39/83 | 0.354 | Fig. 5A |
| *rrf-3(pk1426); daf-10(m79);*  *K12G11.3(RNAi)* | 24.9±1.4 | 33 | +6% | 46/103 | 0.225 | Fig. 5A |
| *rrf-3(pk1426);* *ZC84.3(RNAi); daf-10(m79)* | 24.2±1.2 | 30 | +3% | 43/97 | 0.8509 | Fig. 5A |
| *rrf-3(pk1426);* *F53A3.1(RNAi); daf-10(m79)* | 25.3±1.3 | 33 | +7.7% | 46/82 | 0.2519 | Fig. 5A |
| *rrf-3(pk1426); daf-10(m79); C09H5.2a(RNAi)* | 23.1±1.1 | 28 | -1.7% | 46/82 | 0.5914 | Fig. 5A |
| *rrf-3(pk1426); daf-10(m79); F53B2.2(RNAi)* | 24.9±1.2 | 30 | +6% | 44/80 | 0.3885 | Fig. 5A |
| *rrf-3(pk1426); daf-10(m79); R08E5.4(RNAi)* | 24.1±1.1 | 30 | +2.6% | 40/80 | 0.8662 | Fig. 5A |
| Wild type | 23.0±0.5 | 28 |  | 125/210 |  | Fig. 5C |
|  | 20.3±0.5 | 24 |  | 73/180 |  | Fig. 6A |
|  | 18.8±0.4 | 24 |  | 88/210 |  | Fig. 6B |
|  | 17.5±0.5 | 21 |  | 75/180 |  | Fig. 6D |
|  | 18.3±0.4 | 20 |  | 80/180 |  |  |
|  | 19.4±0.4 | 22 |  | 110/240 |  | Fig. 6E |
|  | 17.1±0.5 | 20 |  | 56/150 |  |  |
|  | 16.2±0.5 | 21 |  | 77/111 |  |  |
| *mct-1/2(RNAi)* | 21.0±0.5 | 24 | -9% | 102/210 | <0.0001 | Fig. 5C |
|  | 22.4±0.6 | 26 | +10% | 64/182 | <0.01 | Fig. 6A |
|  | 19.5±0.4 | 24 | +4% | 88/210 | 0.2645 | Fig. 6B |
|  | 18.6±0.6 | 21 | +6.3% | 64/150 | 0.2091 | Fig. 6D |
|  | 19.4±0.5 | 22 | +6% | 66/180 | 0.0621 |  |
|  | 19.8±0.4 | 22 | +2% | 114/240 | 0.3861 | Fig. 6E |
|  | 19.3±0.5 | 22 | +13% | 69/150 | <0.01 |  |
|  | 14.9±0.4 | 18 | -8% | 74/113 | <0.05 |  |
| *daf-10(m79)* | 29.4±0.7 | 35 | +28% | 86/175 | <0.0001 | Fig. 5C |
|  | 36.7±0.7 | 41 | +32% | 98/180 | <0.0001 |  |
|  | 25.1±0.4 | 28 | +37% | 139/180 | <0.0001 |  |
| *daf-10(m79); mct-1/2(RNAi)* | 22.9±0.5 | 24 | -22% | 134/210 | <0.0001  (<0.01 *^mct-1/2(RNAi)^* ) | Fig. 5C |
|  | 34.1±0.7 | 41 | -7% | 102/180 | <0.05  (<0.0001 *^mct-1/2(RNAi)^* ) |  |
|  | 21.9±0.4 | 26 | -13% | 158/180 | <0.0001  (<0.0001 *^mct-1/2(RNAi)^* ) |  |
| *daf-2(e1370)* | 35.2±0.8 | 44 | +87% | 162/180 | <0.0001 | Fig. 6B |
|  | 47.9±0.9 | 55 | +136% | 122/150 | <0.0001 |  |
| *daf-2(e1370); mct-1/2(RNAi)* | 35.8±0.7 | 41 | +2% | 139/179 | 0.7385 | Fig. 6B |
|  | 47.5±0.9 | 55 | -1% | 119/150 | 0.599 |  |
| *osm-5(p813)* | 28.1±0.6 | 32 | +55% | 58/180 | <0.0001 |  |
|  | 29.2±1.0 | 37 | +44% | 62/150 | <0.0001 | Fig. 6A |
|  | 21.9±0.7 | 27 | +13% | 48/150 | <0.001 |  |
|  | 25.1±0.8 | 31 | +47% | 66/150 | <0.05 |  |
| *mct-1/2(RNAi); osm-5(p813)* | 24.5±0.6 | 27 | -13% | 64/178 | <0.0001 |  |
|  | 29.2±0.9 | 35 | 0% | 72/180 | 0.8057 | Fig. 6A |
|  | 25.1±0.7 | 29 | +15% | 61/180 | <0.01 |  |
|  | 25.1±0.7 | 31 | 0% | 99/180 | 0.6856 |  |
| *eat-2(ad1116)* | 24.3±0.5 | 29 | +39% | 94/150 | <0.0001 | Fig. 6D |
|  | 21.0±0.4 | 23 | +8% | 110/181 | <0.0001 |  |
| *eat-2(ad1116); mct-1/2(RNAi)* | 22.6±0.5 | 26 | -7% | 105/180 | <0.05 | Fig. 6D |
|  | 20.6±0.4 | 23 | -2% | 97/180 | 0.4826 |  |
| *isp-1(qm150)* | 29.9±0.8 | 36 | +54% | 86/108 | <0.0001 | Fig. 6E |
|  | 28.6±0.9 | 35 | +67% | 102/136 | <0.0001 |  |
| *isp-1(qm150); mct-1/2(RNAi)* | 29.9±0.7 | 36 | 0% | 110/135 | 0.8308 | Fig. 6E |
|  | 26.0±0.9 | 31 | -9% | 87/125 | <0.05 |  |
| *rrf-3(pk1426)* | 23.2±0.4 | 27 |  | 123/149 |  | Fig. 5B |
|  | 22.5±0.4 | 25 |  | 108/125 |  |  |
|  | 21.0±0.5 | 25 |  | 88/150 |  |  |
|  | 21.8±0.5 | 26 |  | 97/150 |  | Fig. 6C |
|  | 17.7±0.8 | 23 |  | 81/121 |  |  |
| *rrf-3(pk1426); mct-1/2(RNAi)* | 20.6±0.4 | 25 | -11% | 139/150 | <0.0001 | Fig. 5B |
|  | 20.0±0.4 | 23 | -11% | 134/150 | <0.0001 |  |
|  | 20.1±0.4 | 23 | -4% | 75/150 | <0.05 |  |
|  | 20.5±0.4 | 23 | -6% | 88/150 | <0.05 | Fig. 6C |
|  | 15.0±0.4 | 18 | -15% | 95/120 | <0.001 |  |
| *rrf-3(pk1426); daf-10(m79)* | 34.7±0.7 | 41 | +50% | 125/150 | <0.0001 | Fig. 5B |
|  | 30.8±0.6 | 36 | +37% | 119/150 | <0.0001 |  |
|  | 22.4±0.9 | 29 | +21% | 84/121 | <0.0001 |  |
| *rrf-3(pk1426); daf-10(m79); mct-1/2(RNAi)* | 23.9±0.6 | 28 | -31% | 128/150 | <0.0001  (<0.0001 *^rrf-3;^* *^mct-1/2(RNAi)^* ) | Fig. 5B |
|  | 23.2±0.5 | 26 | -33% | 125/150 | <0.0001  (<0.0001 *^rrf-3;^* *^mct-1/2(RNAi)^* ) |  |
|  | 16.8±0.4 | 21 | -25% | 72/121 | <0.0001  (<0.01  *^rrf-3;^* *^mct-1/2(RNAi)^* ) |  |
| *rrf-3(pk1426); daf-2(e1370)* | 43.4±0.8 | 50 | +106% | 103/150 | <0.0001 |  |
|  | 47.0±0.7 | 51 | +116% | 104/150 | <0.0001 | Fig. 6C |
| *rrf-3(pk1426); daf-2(e1370); mct-1/2(RNAi)* | 39.3±0.8 | 45 | -9% | 100/150 | <0.001 |  |
|  | 47.0±0.8 | 51 | 0% | 101/150 | 0.9169 | Fig. 6C |
| Wild type | 19.0±0.3 | 22 |  | 123/180 |  | Fig. 7K |
|  | 20.6±0.3 | 23 |  | 93/118 |  |  |
| *yhEx64[mct-1::GFP]* | 21.2±0.4 | 24 | +12% | 117/160 | <0.0001 | Fig. 7K |
|  | 21.8±0.3 | 25 | +6% | 108/128 | <0.01 |  |
| *yhEx65[mct-1::GFP]* | 20.1±0.5 | 24 | +6% | 91/150 | <0.01 | Fig. 7K |
|  | 22.2±0.4 | 25 | +8% | 86/134 | <0.01 |  |
| *yhEx66[mct-1::GFP]* | 20.5±0.5 | 24 | +8% | 102/149 | <0.01 | Fig. 7K |
|  | 22.1±0.4 | 25 | +7% | 70/96 | <0.001 |  |
