# Supplementary material for: Genes That Act Downstream of Sensory Neurons to Influence Longevity, Dauer Formation, and Pathogen Responses in Caenorhabditis elegans
Source: PLoS Genet. 2012 Dec 20;8(12):e1003133. doi: 10.1371/journal.pgen.1003133 (PMC3527274; doi:10.1371/journal.pgen.1003133)
Supplement: Table S2 — Statistical analysis of P. aeruginosa (PA14) resistance assay. Survival data within the solid bold lines include sets that were collected in parallel. In particular, within each panel of this table, data sets shaded in the same color were done in parallel. p values for single mutants were calculated against wild type and for double mutants against the corresponding single mutants (immediately above in the table) using the log-rank test (Mantel-Cox method) and the Wilcoxon test. p values in parentheses are marked and explained below. daf-l0: p value against daf-10(m79) mutant. daf-16(RNAi); rrf-3: p value against daf-16(RNAi); rrf-3(pk1426). rrf-3; mct-1/2(RNAi): p value against rrf-3(pk1426); mct-1/2(RNAi). small: p value against wild type on small lawn of PA14. #: These pathogen resistance assays were carried out at 20°C. (DOCX) [file pgen.1003133.s007.docx]

**Table S2. Statistical analysis of *P. aeruginosa* (PA14) resistance assay**

| **Lawn type** | **Strain/treatment** | **Mean lifespan ±s.e.m. (hours)** | **75th percentile** | **% change** | **Number of animals that died/total** | ***P* value*^*^* vs. control**  **(Log-rank)** | ***P* value*^^^* vs. control**  **(Wilcoxon)** | **Figure in text** |
| --- | --- | --- | --- | --- | --- | --- | --- | --- |
| Small | Wild type | 86.5±2.1 | 98 |  | 103/160 |  |  | Fig. 3D |
|  |  | 65.2±1.7 | 70 |  | 111/160 |  |  |  |
|  |  | 71.3±2.2 | 81 |  | 81/120 |  |  |  |
|  |  | 69.2±2.7 | 76 |  | 27/120 |  |  |  |
|  |  | 80.5±2.2 | 89 |  | 91/120 |  |  | Fig. 3B |
|  | *^#^* | 107.5±6.2 | 144 |  | 87/117 |  |  | Fig. S2 |
|  | *^#^* | 90.0±2.2 | 120 |  | 135/148 |  |  |  |
|  | *^#^* | 70.1±4.3 | 96 |  | 45/68 |  |  |  |
|  | *^#^* | 130.3±5.3 | 144 |  | 87/117 |  |  |  |
|  | *^#^* | 137.8±6.5 | 168 |  | 45/111 |  |  |  |
| Small | *daf-10(m79)* | 70.1±1.6 | 73 | -19.0% | 121/170 | <0.0001 | <0.0001 | Fig. 3D |
|  |  | 74.6±3.3 | 101 | +14.4% | 57/160 | <0.05 | 0.1138 |  |
|  |  | 67.0±1.6 | 75 | -5.9% | 101/120 | 0.1502 | 0.3612 |  |
|  |  | 67.7±2.9 | 73 | -2.2% | 43/77 | 0.6019 | 0.1751 |  |
|  |  | 67.7±1.3 | 78 | -15.9% | 92/120 | <0.0001 | <0.0001 | Fig. 3B |
|  | *^#^* | 80.2±3.4 | 120 | -25.4% | 93/115 | <0.01 | <0.05 | Fig. S2 |
|  | *^#^* | 87.1±2.6 | 120 | -3.2% | 140/151 | 0.1473 | 0.0610 |  |
|  | *^#^* | 80.9±2.9 | 120 | +15.4% | 92/112 | <0.05 | <0.05 |  |
|  | *^#^* | 92.6±3.1 | 144 | -28.9% | 123/136 | <0.0001 | <0.0001 |  |
|  | *^#^* | 172.1±17.3 | 192 | +24.9% | 31/114 | <0.05 | <0.05 |  |
| Small | *gcy-35(ok769)* *^#^* | 146.2±6.7 | 192 | +35.9% | 85/110 | <0.001 | <0.0001 | Fig. S2 |
|  | *^#^* | 106.8±3.8 | 144 | +18.7% | 129/143 | <0.001 | <0.01 |  |
| Small | *gcy-35(ok769); daf-10(m79)* *^#^* | 133.4±7.0 | 192 | -8.8% | 77/95 | 0.1784 (<0.0001*^daf-l0^*) | 0.1488  (<0.0001*^daf-l0^*) | Fig. S2 |
|  | *^#^* | 99.8±3.1 | 120 | -6.5% | 138/148 | 0.0947  (<0.001*^daf-l0^*) | 0.3797  (<0.001*^daf-l0^*) |  |
| Big | Wild type | 51.7±0.6 | 68 | -40.2% | 214/300 | <0.0001  ^small^ | <0.0001 ^small^ | Fig. 3D, 4A |
|  |  | 42.9±0.8 | 52 | -34.2% | 105/250 | <0.0001 ^small^ | <0.0001 ^small^ |  |
|  |  | 41.9±0.7 | 51 | -41.2% | 69/150 | <0.0001 ^small^ | <0.0001 ^small^ |  |
|  |  | 43.2±0.8 | 52 | -37.6% | 87/236 | <0.0001 ^small^ | <0.0001 ^small^ |  |
|  |  | 58.2±1.1 | 67 |  | 65/120 |  |  | Fig. S3D |
|  |  | 56.4±0.7 | 68.5 |  | 166/200 |  |  | Fig. 4B |
|  |  | 54.3±0.9 | 65 |  | 96/150 |  |  | Fig. 3C |
|  |  | 32.6±0.6 | 52 |  | 68/120 |  |  |  |
|  |  | 57.2±0.7 | 70 |  | 96/120 |  |  | Fig. S3C |
|  |  | 45.0±0.6 | 57.6 |  | 95/120 |  |  | Fig. S3B |
|  |  | 42.8±0.4 | 56 |  | 112/146 |  |  | Fig. S3A |
| Big | *daf-10(m79)* | 66.8±1.2 | 73 | +29.2% | 119/300 | <0.0001 | <0.0001 | Fig. 3D, 4A |
|  |  | 58.8±4.6 | 70 | +37.2% | 45/250 | <0.001 | <0.05 |  |
|  |  | 71.7±5.7 | 95 | +70.9% | 29/150 | <0.0001 | <0.01 |  |
|  |  | 60.3±2.9 | 85 | +39.7% | 70/250 | <0.0001 | <0.01 |  |
|  |  | 83.3±7.8 | 120.6 | +43.1% | 22/120 | 0.3967 | 0.1511 | Fig. S3D |
|  |  | 78.0±3.4 | 102 | +38.2% | 117/200 | <0.0001 | <0.0001 | Fig. 4B |
|  |  | 82.3±1.4 | 90 | +51.6% | 112/147 | <0.0001 | <0.0001 | Fig. 3C |
|  |  | 43.1±1.4 | 52 | +32.3% | 74/120 | <0.0001 | <0.0001 |  |
|  |  | 92.7±6.0 | 132 | +62.0% | 42/120 | <0.0001 | <0.0001 | Fig. S3C |
|  |  | 72.7±6.2 | 96.1 | +61.4% | 20/120 | <0.0001 | <0.0001 | Fig. S3B |
|  |  | 56.1±3.1 | 56 | 40.0% | 59/150 | <0.0001 | <0.0001 | Fig. S3A |
| Big | *daf-16(mu86)* | 49.2±0.6 | 66 | -4.8% | 242/300 | <0.0001 | <0.0001 | Fig. 4A |
|  |  | 42.6±0.6 | 49 | -0.7% | 130/247 | 0.5735 | 0.0510 |  |
| Big | *daf-16(mu86); daf-10(m79)* | 53.3±0.7 | 65 | +8.3% | 167/300 | 0.0801  (<0.0001*^daf-l0^*) | 0.4489  (<0.0001*^daf-l0^*) | Fig. 4A |
|  |  | 47.2±0.7 | 60 | +10.8% | 99/248 | <0.0001  (0.7728 *^daf-l0^*) | <0.0001  (0.0794 *^daf-l0^*) |  |
| Big | *daf-12(rh61rh411)* | 54.1±0.9 | 66.8 | -7.1% | 79/120 | <0.05 | 0.0586 | Fig. S3D |
|  |  | 61.8±0.8 | 79.5 | +9.6% | 187/200 | 0.1331 | <0.05 | Fig. 4B |
|  |  | 57.3±1.0 | 63.8 | +0.1% | 92/120 | 0.2766 | 0.4713 | Fig. S3C |
|  |  | 46.1±0.8 | 57.3 | +2.4% | 93/120 | <0.05 | <0.01 | Fig. S3B |
|  |  | 45.4±0.5 | 55 | 6.0% | 112/150 | <0.001 | <0.001 | Fig. S3A |
| Big | *daf-10(m79); daf-12(rh61rh411)* | 68.4±1.2 | 73.3 | +26.4% | 101/120 | <0.0001  (0.2041 *^daf-l0^*) | <0.0001  (<0.01 *^daf-l0^*) | Fig. S3D |
|  |  | 81.0±1.1 | 93.2 | +31.1% | 169/200 | <0.0001  (<0.05 *^daf-l0^*) | <0.0001 (<0.0001 *^daf-l0^*) | Fig. 4B |
|  |  | 76.6±1.5 | 91.9 | +33.7% | 101/120 | <0.0001  (<0.05 *^daf-l0^*) | <0.0001  (0.2959 *^daf-l0^*) | Fig. S3C |
|  |  | 68.8±1.2 | 74.8 | +52.8% | 86/120 | <0.0001  (0.0534 *^daf-l0^*) | <0.0001  (0.1863 *^daf-l0^*) | Fig. S3B |
|  |  | 64.7±1.1 | 75 | 51.2% | 116/150 | <0.0001  (<0.001 *^daf-l0^*) | <0.0001  (<0.0001 *^daf-l0^*) | Fig. S3A |
| Big | *rrf-3(pk1426)* | 50.5±0.5 | 56 |  | 177/200 |  |  | Fig. 5E |
|  |  | 41.3±0.5 | 52 |  | 210/250 |  |  |  |
|  | *daf-16(RNAi); rrf-3(pk1426)* | 49.6±0.6 | 56 | -1.8% | 169/200 | 0.2729 | 0.2120 | Fig. 5E |
|  | *rrf-3(pk1426); mct-1/2(RNAi)* | 50.4±0.5 | 56 | -0.2% | 183/200 | 0.9746 | 0.6287 | Fig. 5E |
|  |  | 42.1±0.6 | 52 | +1.9% | 211/250 | 0.4478 | 0.7388 |  |
|  | *rrf-3(pk1426); daf-10(m79)* | 69.1±1.3 | 80 | +37.0% | 119/200 | <0.0001 | <0.0001 | Fig. 5E |
|  |  | 67.6±1.4 | 82 | +63.8% | 132/248 | <0.0001 | <0.0001 |  |
|  | *daf-16(RNAi); rrf-3(pk1426); daf-10(m79)* | 57.8±1.0 | 69 | -16.4% | 171/200 | <0.0001 (<0.0001*^daf-16(RNAi); rrf-3^*) | <0.0001  (<0.0001*^daf-16(RNAi); rrf-3^*) | Fig. 5E |
|  | *rrf-3(pk1426); daf-10(m79); mct-1/2(RNAi)* | 69.4±1.4 | 80 | +0.4% | 141/200 | 0.7802  (<0.0001 *^rrf-3; mct-1/2(RNAi)^*) | 0.9904  (<0.0001 *^rrf-3; mct-1/2(RNAi)^*) | Fig. 5E |
|  |  | 69.6±1.5 | 82 | +3.0% | 166/250 | 0.2129  (<0.0001 *^rrf-3; mct-1/2(RNAi)^*) | 0.4873  (<0.0001 *^rrf-3; mct-1/2(RNAi)^*) |  |
