# Supplementary material for: Genes That Act Downstream of Sensory Neurons to Influence Longevity, Dauer Formation, and Pathogen Responses in Caenorhabditis elegans
Source: PLoS Genet. 2012 Dec 20;8(12):e1003133. doi: 10.1371/journal.pgen.1003133 (PMC3527274; doi:10.1371/journal.pgen.1003133)
Supplement: Table S3 — Sequences of primers used for qRT–PCR analysis. (DOCX) [file pgen.1003133.s008.docx]

**Table S3. Primer sequences**

| **Gene** | **Forward/Reverse** | **Sequence** | **Figure in text** |
| --- | --- | --- | --- |
| K10D11.1/ *dod-17* | Forward | 5’-CACTCACTGTCGCTAACGAAG-3’ | Fig. 1A, Fig. 2A, Fig. 3A, Fig. S1A |
|  | Reverse | 5’-CTACTGTTGGAAGTGGCCACC-3’ |  |
| C32H11.12/ *dod-24* | Forward | 5’-CTGTTACTTTCTACAAACACTTC-3’ | Fig. 1A, Fig. 2A, Fig. 3A, Fig. S1A |
|  | Reverse | 5’-GTTCTGGAGCAATTCAATTTTAC-3’ |  |
| F31F4.15/ *fbxa-72* | Forward | 5’-GTTCCATTTTGACTCATTTACC-3’ | Fig. 1A, Fig. 2A, Fig. S1A |
|  | Reverse | 5’-CATTACATCGTCTCTAATTTTG-3’ |  |
| C05A9.1/ *pqp-5* | Forward | 5’-CTATTCAGGGAATATATTACCC-3’ | Fig. 1A, Fig. S1A |
|  | Reverse | 5’-GAATGCATATGCCTCATAAGAC-3’ |  |
| T21E8.2/ *pqp-7* | Forward | 5’-GTTACACATTGGGAGAAACTGC-3’ | Fig. 1A, Fig. S1A |
|  | Reverse | 5’-GATCGGCAGGGTCAATAGTTTTG-3’ |  |
| C17H12.6 | Forward | 5’-GGATACAATGATTTGATTACTG-3’ | Fig. 1A, Fig. 2A, Fig. S1A |
|  | Reverse | 5’-GATGGAGAACGACTTGAAACAC-3’ |  |
| C32H11.4 | Forward | 5’-GGGTGATAAGCATTCATACTTC-3’ | Fig. 1A, Fig. 2A, Fig. 3A, Fig. S1A |
|  | Reverse | 5’-CTCCAATTTTGTTTGAACAACG-3’ |  |
| F55G11.2 | Forward | 5’-CCATCGCTGTCACTAACGAAG-3’ | Fig. 1A, Fig. 2A, Fig. S1A |
|  | Reverse | 5’-GTGATGATGGAAGTGGCCACC-3’ |  |
| F55G11.3 | Forward | 5’-CCAGCCTCAAAGTTCACCATTGC-3’ | Fig. 1A, Fig. 2A, Fig. S1A |
|  | Reverse | 5’-GATGGTGGAAGTGGCCACCAAAC-3’ |  |
| K10C2.3 | Forward | 5’-CCAACTCACGATGTCTTCCTTC-3’ | Fig. 1A, Fig. 2A, Fig. S1A |
|  | Reverse | 5’-GTCAAGGTAAGTTTGAATGTAC-3’ |  |
| K11H12.4 | Forward | 5’-CAATGACACCACAACCAGTAGG-3’ | Fig. 1A, Fig. S1A |
|  | Reverse | 5’-CAGAAAAGGTTGGATCTTGTGC-3’ |  |
| T19D12.4 | Forward | 5’-CGTGGCTCAATACAAATACGG-3’ | Fig. 1A, Fig. S1A |
|  | Reverse | 5’-CAACAACGTCGCTGCTGAAAG-3’ |  |
| T24B8.5 | Forward | 5’-CAGATGTTAGAAGTGGTTGTCG-3’ | Fig. 1A, Fig. S1A |
|  | Reverse | 5’-GACCGAGCTTGTCTTCGCATAC-3’ |  |
| T24C4.4 | Forward | 5’-CCAGTTGGATGATGTTCATCTC-3’ | Fig. 1A, Fig. 3A, Fig. S1A |
|  | Reverse | 5’-GTATACCTTGTGTGACGCGTGC-3’ |  |
| Y49G5A.1 | Forward | 5’-CAGTGGATGTGGAGGAAACAAG-3’ | Fig. 1A, Fig. 2A, Fig. S1A |
|  | Reverse | 5’-CCCGATTGATCATATCCAGTAC-3’ |  |
| Y69A2AR.25 | Forward | 5’-CCGACAGCATCATATGGCACGG-3’ | Fig. 1A, Fig. S1A |
|  | Reverse | 5’-GGCAAGAATCCAAGACGCATTG-3’ |  |
| ZK6.11 | Forward | 5’-CAATATGCAGACAACACCTTTC-3’ | Fig. 1A, Fig. S1A |
|  | Reverse | 5’-CAACTTTTCCTAGTGCTTGCAG-3’ |  |
| K12G11.3/ *dod-11* | Forward | 5’-GAACAAGCTGTTGAATACGTCAG-3’ | Fig. 1B, Fig. S1B |
|  | Reverse | 5’-GAAAGTAACCTTTGAATCCTTTG-3’ |  |
| C09H5.2 | Forward | 5’-CGGAACTTTGACCAAGAATTG-3’ | Fig. 1B, Fig. S1B |
|  | Reverse | 5’-CTGGTCTTGCGCTGTTATATG-3’ |  |
| Y19D10A.12/ *mct-1/2* | Forward | 5’-CAGATCAGTTTGGATCCACATC-3’ | Fig. 1B, Fig. S1B |
|  | Reverse | 5’-CAGTGGTCCAGCAAGATAATAG-3’ |  |
| F56A4.3 | Forward | 5’-CTTCACTATTCGAGGATTCGGAG-3’ | Fig. 1B, Fig. S1B |
|  | Reverse | 5’-CGTAGTCGAACCGAATGTCTTCG-3’ |  |
| ZK105.5 | Forward | 5’-GAGAGTTTTGTTTTCTTACAC-3’ | Fig. 1B, Fig. S1B |
|  | Reverse | 5’-GCCGGATCGCTCGACATAAAG-3’ |  |
| C08A9.1/ *sod-3*C08A9.1C08A9.1 C08A9.1 | Forward | 5’-CTATCTTCTGGACCAACTTGG-3’ | Fig. S5A |
|  | Reverse | 5’-GCAAGTTATCCAGGGAACCG-3’ |  |
| K11G9.6/ *mtl-1* | Forward | 5’-ATGGCTTGCAAGTGTGACTGCAAAAACAAGC-3’ | Fig. S5A |
|  | Reverse | 5’-TTAATGAGCCGCAGCAGTTCCCTGGTGTTGATGGG-3’ |  |
| C07G3.2 / *irg-1* | Forward | 5’-GATCTTGTTCCGTACCCATGG-3’ | Fig. 3A |
|  | Reverse | 5’-GCTTTGTCAAGACCAATTCCC-3’ |  |
